# Trace metal exposure is associated with increased exhaled nitric oxide in asthmatic children

**DOI:** 10.1186/s12940-016-0173-5

**Published:** 2016-09-01

**Authors:** Krystal J. Godri Pollitt, Caitlin L. Maikawa, Amanda J. Wheeler, Scott Weichenthal, Nina A. Dobbin, Ling Liu, Mark S. Goldberg

**Affiliations:** 1 Department of Environmental Health Sciences, School of Public Health and Health Sciences, University of Massachusetts, 149D Goessman Lab, 686 North Pleasant Street, Amherst, MA 01003 USA; 2 Health Canada, Air Health Science Division, Ottawa, ON Canada; 3 Menzies Institute for Medical Research, University of Tasmania, Private Bag 23, Hobart, TAS 7000 Australia; 4 Department of Epidemiology, Biostatistics, and Occupational Health, McGill University, Montreal, QC Canada; 5 Health Canada, Population Studies Division, Ottawa, ON Canada; 6 Department of Medicine, Division of Clinical Epidemiology, Research Institute, McGill University Health Centre, McGill University, Montreal, Canada

**Keywords:** Children, Air pollution, Trace metals, Exposure, Asthma, Lung inflammation, Exhaled nitric oxide, Urban environment, Petroleum refinery, Panel study, Particulate matter

## Abstract

**Background:**

Children with asthma experience increased susceptibility to airborne pollutants. Exposure to traffic and industrial activity have been positively associated with exacerbation of symptoms as well as emergency room visits and hospitalisations. The effect of trace metals contained in fine particulate matter (aerodynamic diameter 2.5 μm and lower, PM_2.5_) on acute health effects amongst asthmatic children has not been well investigated. The objective of this panel study in asthmatic children was to determine the association between personal daily exposure to ambient trace metals and airway inflammation, as measured by fractional exhaled nitric oxide (FeNO).

**Methods:**

Daily concentrations of trace metals contained on PM_2.5_ were determined from personal samples (*n* = 217) collected from 70 asthmatic school aged children in Montreal, Canada, over ten consecutive days. FeNO was measured daily using standard techniques.

**Results:**

A positive association was found between FeNO and children’s exposure to an indicator of vehicular non-tailpipe emissions (8.9 % increase for an increase in the interquartile range (IQR) in barium, 95 % confidence interval (CI): 2.8, 15.4) as well as exposure to an indicator of industrial emissions (7.6 % increase per IQR increase in vanadium, 95 % CI: 0.1, 15.8). Elevated FeNO was also suggested for other metals on the day after the exposure: 10.3 % increase per IQR increase in aluminium (95 % CI: 4.2, 16.6) and 7.5 % increase per IQR increase in iron (95 % CI: 1.5, 13.9) at a 1-day lag period.

**Conclusions:**

Exposures to ambient PM_2.5_ containing trace metals that are markers of traffic and industrial-derived emissions were associated in asthmatic children with an enhanced FeNO response.

**Electronic supplementary material:**

The online version of this article (doi:10.1186/s12940-016-0173-5) contains supplementary material, which is available to authorized users.

## Background

Exacerbation of paediatric asthmatic symptoms as well as increased emergency room visits and hospitalisations have been associated with exposure to ambient particulate matter (PM) [1–8]. The incidence of these exacerbations and hospitalisations has been found to be elevated amongst asthmatic children residing in communities with dense traffic and industrial activity [4, 9, 10], attributed to the emission of trace metals released by these myriad sources [11–13]. Exposure to trace metals from traffic emissions may be derived from non-tailpipe releases (e.g., wearing of brakes and tires) or from the resuspension of road dust. Abrasion of brake pads in vehicles is characterised by barium, copper and antinomy [14, 15] while zinc is attributable to tire wear in microenvironments primarily influenced by traffic emissions [16]. Crustal elements are typically derived from road (calcium, magnesium, iron) and soil dust (aluminium, potassium, silica) [14, 17]. Industrial sources release a wide range of trace metals. Combustion of residual oil for heating in residential buildings is a major source of nickel and vanadium [18]. Nickel and vanadium are also associated with petroleum refinery activities, in addition to aluminium, arsenic, chromium, iron, sulphur and zinc [19–24]. Emissions from industrial petroleum refinery operations have been shown to adversely impact the air quality in a number of major Canadian cities, including Montreal, Edmonton and Halifax [17].

In recent cross-sectional studies, children’s exposure to these industrial- and traffic-derived trace metals and the implications on their respiratory health has been investigated. In Baltimore, ambient zinc from fine particulate matter (particles with an aerodynamic diameter of 2.5 μm and lower; PM_2.5_) was found to be associated with paediatric asthmatic hospitalisations and emergency room visits [11]. Children with respiratory disease living in California were shown to experience increased hospitalisation rates following exposure to increased iron and zinc [25]. In New York City, asthmatic children from the Columbia Center for Children’s Environmental Health birth cohort who had increased exposure to nickel and vanadium from PM_2.5_ were found in longitudinal analyses to suffer from increased wheeze [13]. A cross-sectional analysis of this New York City cohort, among children who were nine or 11 years of age with valid measurements of fractional exhaled nitric oxide (FeNO) showed positive associations with concentrations of ambient iron, nickel and vanadium [12].

FeNO has been hypothesised to be an indicator for up-regulation of eosinophilic airway inflammation [26]. While the actual mechanisms that are indicated by FeNO are not known, this non-invasive measurement has been validated against other markers of inflammation, including IgE levels and blood eosinophils [27–29]. FeNO is often tested in children to evaluate the presence and severity of asthma [30]. Allergic asthmatics often exhibit elevated FeNO that increases after allergen exposures [31].

Two previous studies conducted in Montreal, Canada, suggested that the respiratory health of children might be affected by living in proximity to heavy industry and traffic [32, 33]. Hospitalisation for respiratory illness was 25 % higher in children 2–4 years of age residing in the area around the two petroleum refineries compared to those residing in other regions of Montreal. This finding was the motivation for the present study that was designed as a panel study among asthmatic children who lived near these petroleum refinery facilities. The objective was to determine whether acute, personal exposures to selected air pollutants were associated with selected health endpoints [32–34]. In a previous paper, we measured daily variations in personal exposure to fine particulates and estimated oxidative burden from samples of PM_2.5_ [35]. We found that personal PM_2.5_ exposure characterised by enhanced oxidative burden was associated with an increased eosinophilic airway inflammation, measured as FeNO. In the present paper, we extend these analyses of this dataset to make use of concentrations of trace metals and again relate this to daily changes in FeNO.

## Methods

### Population

Children diagnosed with asthma, between the ages of 8 and 13, were the target population for this study. Children with physician-diagnosed cases of asthma were identified from records at a paediatric asthma clinic in a tertiary-care adult Montreal hospital (Maisonneuve-Rosemont), the main centre for referrals for childhood asthma in the area (details described previously [34]). To meet sample size requirements, additional children were recruited in the latter half of the study from local schools. Parents of children at these schools were encouraged to contact the study centre if a doctor had diagnosed their child with asthma.

The diagnosis of asthma for children was confirmed by respirologists for participants recruited from the asthma clinic. Among those enrolled from the schools, guardians confirmed the presence of asthma in their children through a series of questions related to whether a doctor had made a diagnosis of asthma. Parents also confirmed they met the following eligibility criteria: being a non-smoker and not exposed to environmental tobacco smoke at home; fluent in French or English, capable of giving informed consent; and capable of participating in the study. Interested families that met the above criteria were given a detailed explanation of the study during a home visit.

During the 10-day study period, children’s homes were visited daily between 4 pm and 6 pm to take FeNO measurements and give daily activity questionnaires. The consecutive 10-day observation period took place between October 2009 and April 2010.

The children’s guardians filled out questionnaires regarding their home and health at the beginning of the study. Health questionnaires elicited demographic characteristics, child’s age when diagnosed with asthma, typical respiratory symptoms, medication use, existing allergies (presence of eczema, hay fever, and allergies) and the presence of parental asthma, smoking, or allergies. Home questionnaires collected information on the type of home (e.g., detached), pets, number of residents, cooking devices, and ventilation and heating source.

### Ethics statement

Written, informed consent given by children’s guardians was necessary for their child to take part in the study, and the children gave verbal assent to participate. Research ethics boards at Health Canada, the McGill University Health Centre, Direction de santé publique de Montréal and Hôpital Maisonneuve-Rosemont approved the study.

### Personal exposure to metals

Children carried personal monitors capable of measuring temperature, relative humidity and particulate pollutants in a backpack on wheels. These rolling backpacks, weighing approximately 7 kg, were kept nearby throughout the study (e.g., placing the backpack on a nearby chair while at rest), although no direct methods were applied to assess compliance. If the children were in one location for an extended period of time (i.e., at school, playing sports, sleeping), they were instructed to keep the backpack in the same environment close to them with the sampling inlet facing up. Daily activity (e.g., hours spent out- and indoors, sports activities) and medication use (i.e., beta-agonists and corticosteroids) was recorded in a daily diary.

A continuous PM_2.5_ monitor (pDR-1200; ThermoScientific,) with an after-filter (PEMs, Chempass System R&P / Thermo; using Teflon filters) was used to collect 24-h samples over the 10-day observation period. The PM_2.5_ monitor was operated at a flow rate of 4 l per minute. Flow rate and samples were checked during daily home visits in addition to replacing equipment batteries. We gravimetrically measured the mass concentration of PM_2.5_. The gravimetric analysis was performed using the United States Environmental Protection Agency method outlined in the Quality Assurance Guideline Document 2.12 [36]. The corresponding continuous and gravimetric measurements of samples were deemed invalid if the end flow rate of the PM_2.5_ sampler was found to be 20 % above or below the target. Samples deployed for more than 30 h or less than 18 h were also deemed invalid.

The concentration of selected metals using non-destructive X-Ray fluorescence technique (RTI Laboratories, Research Triangle Park, NC) was conducted. The X-Ray fluorescence protocol followed the EPA method IO-3.3 (EPA 625/R-96/010). All valid samples having a mass greater than 5 μg were analysed for sulphur, while samples having a mass greater than 10 μg were analysed for 33 elements, including sulphur. Field blanks and duplicates comprised approximately 10 % each of all deployed samples.

Temperature and relative humidity were measured using a Hobo sensor (Hobo U10, Onset Computer Corp., Hoskin Scientific Ltd.) placed in an outside pocket of the backpack.

### Measurement of fractional exhaled nitric oxide

FeNO was measured following standardised protocols recommended by the American Thoracic Society and the European Respiratory Society [26, 37] using the NIOX MINO monitor (Aerocrine). Children initially inhaled through the monitor to assess total lung capacity. A slow vital capacity manoeuvre was then performed by requesting children to exhale over a six second period such that an exhalation rate of 50 ± 5 mL/s was achieved. Two replicate measurements were conducted to calculate the FeNO value. If measurements were not within 10 % or 3 μL/L, children were asked to perform a third measurement. We asked children to refrain from eating one hour prior to the FeNO measurements and measured their body temperature prior to measurements to ensure they were not ill. FeNO was calculated as the average of a minimum of two exhalations. Values of FeNO below the minimum detection limit of 5 μL/L were substituted as 2.5 μL/L.

### Statistical methods

The association between FeNO and personal exposures to trace metals over the 10-day observation period was assessed using a linear mixed model with restricted maximum likelihood estimation. To normalise residuals, measurements of FeNO were transformed using a natural logarithm. We accounted for within-subject serial autocorrelation in all models using the following: a first-order autoregressive correlation structure, a random effects indicator for each child, and an indicator for the day of measurement (1–10).

The relationship between personal exposures to the trace metals (aluminium, arsenic, barium, chromium, copper, iron, magnesium, manganese, nickel, antinomy, strontium, vanadium, zinc), as well as sulphur, expressed per unit mass of PM_2.5_ (pg/μg) and the natural logarithm of FeNO were evaluated separately as fixed effects. We used three time periods for exposures to the metals, including 24 h prior to the FeNO measurement (referred to as the 0-day lag), the day before the FeNO measurement (24–48 h average, 1-day lag), and two days before the FeNO measurement (48–72 h average, 2-day lag).

We postulated the following variables were potential confounders: personal measurements of average daily personal temperature and relative humidity; age; sex; corticosteroid use; use of rescue medication (short acting beta-agonists); presence of allergies; occurrence of an asthma attack in the first year of life; eczema before the age of two; and parental asthma. Eczema before the age of 2 years was selected as a potential confounder given the prevalence of this skin disorder in infants who go on to develop allergic asthma later in childhood [38]. We included asthma attack in the last 12 months as a potential covariate related to asthma severity and increased FeNO. While the role of exposures to air pollution on the onset of eczema in infants is not clear, asthma attacks can be exacerbated in children residing in regions with elevated air pollutant concentrations [39]. Use of beta-agonists was defined as a binary variable (no reported use or use on the monitoring day) while corticosteroid use was defined as no reported use, irregular use or regular use (medication use on a minimum of eight of the 10 monitoring days).

A “baseline model” was developed, including subject and time variables, *a priori* potential confounding factors (24-h averaged ambient temperature and sex) and 24-h averaged trace metal concentration. This model was used to assess the extent of confounding due to other personal variables which were evaluated in subsequent analyses: medication use (corticosteroids, short acting beta-agonist); presence of allergies; occurrence of an asthma attack in the first year of life; eczema before the age of two; and parental asthma. These personal variables were added individually to the baseline model (i.e., a random effect for subject, time variables, and *a priori* potential confounding factors of sex, average personal daily temperature) to assess the effect on FeNO.

The “final model” included variables observed to change by 10 % or more the effect of the logarithm of FeNO per interquartile range (IQR) in concentrations of metals. Potential confounding variables were added to the baseline model to build the final model. Personal variables found to meet this criterion were diagnosis of allergies, occurrence of an asthma attack in the previous 12 months, use of short acting beta-agonists, use of corticosteroids, parental asthma and eczema before the age of two (see Additional file 1: Tables S3-S5 for the results of the models). In the text, all results quoted are for these final models and these final models were also used to determine whether medication use and presence of allergies were effect modifiers.

We evaluated the children’s medication use (corticosteroids or short acting beta-agonists) and allergies (fur, dust, pollen, mould) as possible effect modifiers by including an interaction term with personal trace metal exposure concentration. Models with and without this interaction term were compared with the likelihood-ratio statistic using full maximum likelihood. The restricted maximum likelihood approach was used to estimate model parameters once a potentially important interaction was found to be present, and the interpretation of the results was based on these models, as parameter estimates and associated standard errors are unbiased.

To verify that the data met the assumptions of residual autocorrelation and normality of the residuals of the random effects, residual diagnostics were assessed. Possible non-linear associations between FeNO and trace metal concentrations were evaluated using natural cubic splines with two to five degrees of freedom.

## Results

This study included 70 children, allowing for 700 total possible observations. Missing FeNO measurements decreased the total number of samples by 25. The median FeNO value was 17.7 μL/L (IQR = 25.2) in the full dataset. Personal sampler flow problems, metal measurement errors or low PM_2.5_ mass loading on filters required additional observations to be omitted from analysis. The median FeNO value was also evaluated for all filters with available trace metal measurements (Additional file 1: Table S1). FeNO ranged from a median value of 16.3 μL/L (17.3) for available antinomy measurements to 24.5 (30.7) μL/L for available strontium measurements.

Table 1 shows selected characteristics of the 70 children who participated in this study. The children were predominately Caucasian (69 %) and were boys (70 %). Approximately half of the children used corticosteroids, with 18.6 % of all subjects using corticosteroids regularly (>8 days of the 10 day monitoring period). Seventy percent of the children had been diagnosed previously with an allergy (mould, dust, fur, pollen).

**Table 1:** Distribution of selected characteristics of the children

Demographics	
Median age (range), in years	9.9 (8 – 13)
Gender, no. (%)	
Males	49 (70)
Females	21 (30)
Race, no. (%)	
Caucasian	48 (69)
Black	12 (17)
Other	10 (14)
Health status	
Allergies, no. (%)	49 (70)
Hay fever, no. (%)	17 (25)^a^
Eczema before age 2, no. (%)	21 (31)^b^
Asthma attack in previous 12 months, no. (%)	32 (46)
Parental asthma, no. (%)	40 (57)
Medication use during monitoring	
Corticosteroids, no. (%)	34 (49)
Rescue medication (short acting beta-agonist), no. (%)	23 (33)
Median FeNO (IQR), in μL/L	26.6 (23.5)

Note: ^a^ missing information for 3 children

^b^ missing information for 2 children

Table 2 shows the distribution of daily personal exposures to ambient trace metals and meteorological parameters. The median daily personal exposure temperature was 21.1 °C (IQR = 2.0 °C) and relative humidity was 46.7 % (22.1 %) over the October to April monitoring period. Medians and associated IQRs for 24 h integrated traffic-derived trace metal exposures were: barium, 8.66 (9.42) ng/m^3^, copper, 6.44 (10.4) ng/m^3^, and antimony, 22.8 (15.3) ng/m^3^. The median concentrations (IQR) of metals derived from soil and road dust were: aluminium, 49.4 (54.2) ng/m^3^, iron, 53.2 (59.4) ng/m^3^, and magnesium, 11.7 (15.1) ng/m^3^. Concentrations of aluminium and iron were higher during the autumn and spring months as compared to the winter: the median (IQR) concentrations of aluminium were 51.3 (45.3), 27.1 (28.1) and 56.7 (56.7) ng/m^3^ in the autumn, winter and spring, respectively. The median (IQR) concentrations of iron were 56.9 (49.7), 33.0 (25.7) and 57.5 (54.2) ng/m^3^ in the autumn, winter and spring, respectively. No change was found in personal magnesium exposure concentrations across seasons (data not shown).

**Table 2:** Daily personal exposures to trace metals

Exposure	N	Mean (SD)	Median	Interquartile Range	Minimum/Maximum	Minimum Detection Limit
Trace Metals (ng/m^3^)						
Al	211	61.4 (45.7)	49.4	54.2	0.24/267	0.0049
As	86	1.01 (0.67)	0.91	0.79	0.12/3.12	0.0003
Ba	172	10.3 (7.88)	8.66	9.42	0.15/36.6	0.0015
Cr	186	3.45 (4.24)	2.08	2.98	0.01/28.8	0.0003
Cu	210	11.9 (17.2)	6.44	10.4	0.53/128	0.0003
Fe	215	68.3 (48.9)	53.2	59.4	8.22/353	0.0003
Mg	182	14.6 (10.7)	11.7	15.1	0.25/50.9	0.0015
Mn	207	2.68 (2.68)	2.06	2.21	0.08/28.8	0.0003
Ni	176	1.03 (1.06)	0.77	0.90	0.01/8.71	0.0002
S	217	271 (164)	231	179	41.5/1151	0.0010
Sb	58	22.8 (15.3)	19.0	23.4	2.23/68.4	0.0167
Sr	110	2.80 (3.04)	1.68	3.22	0.04/13.0	0.0004
V	131	1.97 (1.49)	1.61	2.18	0.06/6.32	0.0004
Zn	211	23.5 (27.2)	15.5	18.2	1.08/238	0.0009
Ambient temperature (°C)	215	21.1 (1.53)	21.0	2.00	16.0/26.0	
Relative humidity	215	46.7 (13.0)	43.9	22.1	21.0/68.4	

The minimum detection limit (ng/m^3^) for the measurements was calculated using a flow rate of 4 LPM for 24 h filter samples

The metals-related to petroleum refinery emissions were found to have the following median (IQR) concentrations over the complete study period: nickel, 0.77 (0.90) ng/m^3^, vanadium, 1.61 (2.18) ng/m^3^ and sulphur, 231 (179) ng/m^3^. Personal concentrations of nickel and vanadium were the highest in the spring months (data not shown).

Additional file 1: Table S2 shows Spearman correlation coefficients (r) between the metals. Aluminium, iron and magnesium were all correlated. Metals that are characteristic of petroleum refinery emissions (sulphur, nickel and vanadium) were also correlated (r ~ 0.45–0.56). Iron and aluminium were further found to be correlated with both nickel and vanadium (r ~ 0.48–0.55). We found lower correlations (r <0.26) across the metals released from non-tailpipe vehicle emissions (barium, copper, antimony).

Children who did not use any medications to manage asthma had a mean FeNO of 26.5 μL/L in this study. A decreased mean FeNO was found for children who regularly used corticosteroids (16.4 μL/L) while children who irregularly used this medication had a mean FeNO of 37.4 μL/L. A higher mean FeNO was exhibited by children who had an allergy (29.9 μL/L) as compared to children with no allergies (19.0 μL/L).

No deviations from linearity, using natural cubic spline functions with 3° of freedom, were found for any of the personal exposures to the metals (data not shown). Consequently, we present the associations between FeNO and personal exposures to the trace metals as the mean percent change in FeNO for an increase in the IQR of each metal. In the following text, we only quote the results for the final, adjusted models, but the Additional file 1 contains additional details of the various models considered.

**Fig. 1:**
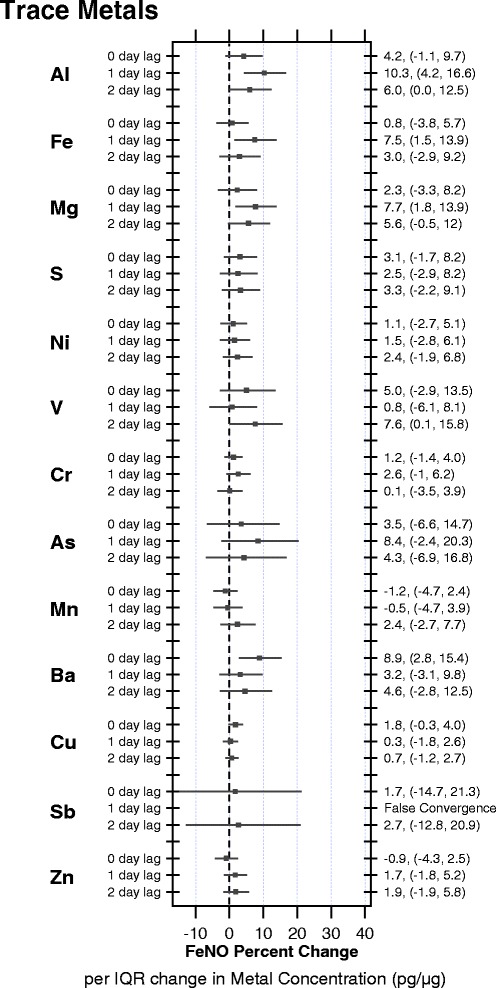
Percent change in FeNO per IQR change (95 % confidence limit) in concentrations of metals derived from personal exposures to PM2.5 over a 0-, 1- and 2- day lags across all children

The main results in Fig. 1 present the fully adjusted models for the association between FeNO and the metals across all children (numerical values are detailed in the Additional file 1: Table S6). Figure 1 shows that across all metals, barium, a metal derived from non-tail pipe emissions, was positively associated with FeNO at the 0–day lag (past 24 h); namely, a 8.9 % increase in FeNO (95 % confidence interval (CI): 2.8–15.4) per increase in IQR of barium (IQR = 0.8 pg/μg) for all children. We further found a suggestion of a positive association for road dust at the 1-day lag: 7.5 % (95 % CI: 1.5, 13.9) per IQR increase of iron (IQR = 4.1 pg/μg) and 7.7 % (95 % CI: 1.8, 13.9) per IQR increase of magnesium (IQR = 1.2 pg/μg) across all children. FeNO was found to increase 10.3 % (95 % CI: 4.2, 16.6) per IQR of aluminium exposure for all children, likely derived from soil dust, at the 1-day lag. At the 2-day lag period, personal exposure to aluminium (6.0 % per IQR change, 95 % CI: 0.0, 12.5) and vanadium (7.6 % per IQR change, 95 % CI: 0.1, 15.8) were suggested to be positively associated with FeNO for all children.

Children who did not use any medications to manage asthma had a mean FeNO of 26.5 μL/Lin this study. A decreased mean FeNO was found for children who regularly used corticosteroids (16.4 μL/L) while children who irregularly used this medication had a mean FeNO of 37.4 μL/L. Personal metal exposure was not associated with FeNO at 0-, 1- and 2-day lag periods among children taking asthma medications (corticosteroids, beta-agonists) (Additional file 1: Table S7).

**Fig. 2:**
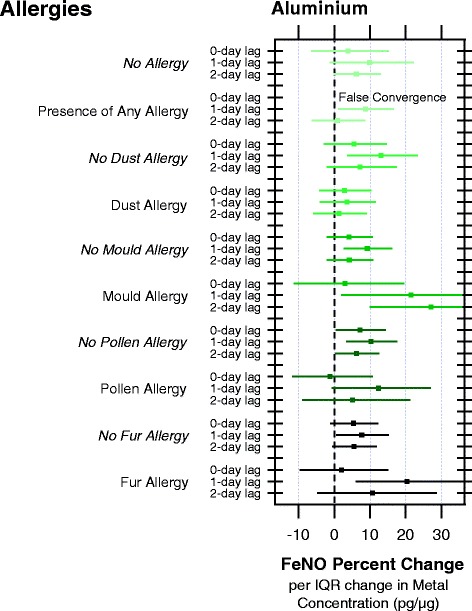
Percent change in FeNO per IQR change (95 % confidence limit) in aluminium concentrations from personal exposures to PM2.5 over a 0-, 1- and 2- day lags as modified by the presence of allergies (dust, mould, pollen, fur)

We assessed interactions with medications and allergies (Additional file 1: Table S8 detail the complete list of trace metal evaluated). No interactions were found with medications but there was a suggestion of an interaction with some allergies. Notably, we found a higher mean FeNO was exhibited by children who had an allergy (29.9 μL/L) as compared to children with no allergies (19.0 μL/L) (Fig. 2). The association between trace metal exposure and FeNO response was evaluated across subgroups of children with and without the presence of allergies. A suggestion of an enhanced FeNO response to aluminium exposures was found at the 1-day lag in children without an allergy to mould (9.2 %, 95 % CI: 2.8, 16.8) or fur (7.4 %, 95 % CI: 0.8, 15.1) but also for children with an allergy to mould (21.6 %, 95 % CI 2.2, 44.6) or fur (20.4 %, 95 % CI 6.2, 36.5). No associations of other trace metal exposures with FeNO according to presence of allergies (mould, dust, fur or pollen) were found for any of the lag periods evaluated (Additional file 1: Table S8).

## Discussion

This is the first panel study to consider exposures to trace metals of paediatric asthmatics and FeNO, a suggested marker of eosinophilic airway inflammation. Our results suggest exposure to metals primarily derived from non-tailpipe vehicular emissions (barium) as well as soil and road dust (iron, magnesium, aluminium) were positively associated with an increased FeNO response in children with asthma. The FeNO response associated with barium was found to be the greatest within 24-h of exposure while iron, magnesium and aluminium exposure was positively associated with elevated FeNO on the day following the exposure (*i.e*., 1-day lag) and vanadium was associated with increased FeNO on the second day following the exposure (*i.e.,* 2-day lag). In our previous paper on these children, we did not find that mass concentrations of PM_2.5_ were associated with changes in FeNO [35].

Four studies have been published assessing trace metal exposure of children and respiratory responses. Two of these studies [12, 13] were conducted in the Bronx, a borough of New York City characterised by dense traffic and a number of industrial facilities. Whilst children assessed in these studies were not limited to asthmatics, findings are similar to our results. Trace metal exposures were estimated using filter samples collected at a central monitoring site. Patel et al., in a longitudinal analysis of a birth cohort, evaluated infants (aged 3–23 months) and used 3-month moving averaged nickel, vanadium and zinc concentration to estimate exposure. The authors found nickel and vanadium, derived from residential heating oil emissions, to be positively associated with wheeze [13]. Rosa et al. made use of cross-sectional analyses at ages nine and 11 in this cohort, and found that FeNO increased with increasing concentrations of iron, nickel and vanadium determined as the average three 24-h filters sampled at three day intervals [12].

The two other studies were also conducted in the United States. A cross-sectional study in California made use of central monitoring sites for estimating exposure, and a positive association was found between respiratory related hospital visits as well as morbidity in a cohort of asthmatic children (<19 years) and trace metals, including copper, iron, and to a lesser extent, zinc [25]. Ambient zinc measured at a central monitoring site was also reported to be positively associated with hospitalisations for asthmatic children (<19 years) from a cross-sectional study in Baltimore [11].

We found positive associations between PM_2.5_ trace metals and percent change in FeNO across all children at the 0-day lag for barium, 1-day lag for aluminium and iron, as well as at the 2-day lag for vanadium. Given the lack of consistency across all lags, it is possible the identified associations were due to chance. No other panel studies have investigated the lag effect of PM trace metal exposure on FeNO. Few panel studies have evaluated the effects of PM mass concentration on FeNO. Positive associations between FeNO, collected daily over 10 consecutive days, and PM_2.5_ mass concentrations were reported for a panel study of children living in Seattle, Washington (aged 6–13) [40]. Enhanced FeNO levels were observed for *1-day averaged* PM_2.5_ mass concentrations measured inside and outside the children’s home using personal monitors as well as at central monitoring sites. Further evaluation of this panel from Seattle found positive associations between *1-h averaged* PM_2.5_ mass concentrations collected from the central monitoring sites for up to 12 h post-exposure [41]. American and Mexican asthmatic children living in proximity to the border were also reported to experience small FeNO increases with elevated *3-day averaged* nitrogen dioxide and PM_2.5_ mass concentrations measured in school playgrounds [42].

An increased FeNO response has been previously reported in asthmatic children not taking medications and compared to regular corticosteroid users [41]. In this study, we found similar results. However, when we stratified by medication use, we found no difference in FeNO increase per IQR increase of PM_2.5_ trace metals for children who did not use any medications compared to children that regularly used corticosteroids. These results suggest that the FeNO response induced by the trace metal fraction of PM is not modified by use of asthma medications.

Suggestions of enhanced exacerbations of asthmatic symptoms in children exposed to ambient pollutants have been reported previously for exposures to nitrogen dioxide [43], black carbon [44], formaldehyde and acetaldehyde [45] as well as trace metals (vanadium and iron from residential oil heating) [12]. In contrast to these findings, we did not observe increased FeNO with exposure to PM_2.5_ trace metals for children with allergies. Rather, a positive association between FeNO response and aluminium was found for children without allergies. Only increased exposure to aluminium was associated with enhanced FeNO for children with allergies to mould. It should be noted that when stratifying by presence of allergy, number of children categorised without an allergy to mould was greater than with an allergies. Sample size may have limited our ability to full evaluate the presence of an allergy as a potential effect modifier.

Our results showed a correlation between nickel and vanadium. These metals are known to be reliable tracers for industrial emissions released from oil combustion and petroleum refineries [17, 19, 46]. While aluminium, magnesium and iron are commonly derived from soil and road dust [23, 24, 46], personal exposure concentrations were also correlated between aluminium and iron with nickel and vanadium. Aluminium is used as a catalyst coating in fluidised bed reactors of petroleum refineries [17, 21]; however, emissions are not released during routine operation [21, 22]. Undeveloped land susceptible to soil erosion and high heavy-duty vehicle traffic in the area surrounding the refineries are likely attributable to the relationship identified between the petroleum and dust sources.

Source apportionment analysis was not conducted on the trace metal measurements collected in this study. We have thus inferred sources from previous studies of the chemical characterisation of emission sources. We acknowledge that our interpretation of these personal concentrations of trace metals may not be accurate and indoor sources of trace metals may have also contributed to the children’s exposure. We note, however, that a PM_2.5_ source apportionment study in Montreal by Jeong et al. confirmed the city is strongly impacted by petroleum combustion and refinery operations [17]. Comparison of emissions from petroleum facilities in Montreal, Halifax and Windsor suggested similar PM_2.5_ mass concentration contributions (annual PM_2.5_ average of 1.2 μg/m^3^) [17]. In contrast, no emissions from this industrial sector were identified in the cities of Toronto or Vancouver.

All of the published studies evaluating trace metal exposure of asthmatic children estimated exposure through measurements conducted at central monitoring sites. Use of personal monitoring equipment in the current study was a strength, as we were able to report more accurate estimates of a child’s personal exposure. Longitudinal personal monitoring is another strength of this study’s design. Repeated follow-up over ten consecutive days enabled detailed analysis of short-term respiratory effects.

Limitations of our study include the use of FeNO measurements to infer airway inflammation. FeNO is a subclinical biomarker for inflammation and while standardised, cannot be relied upon as the sole identifier for asthmatic exacerbations or airway inflammation [26]. FeNO rises in cases of eosinophilic airway inflammation but a response may not be detected for other types of inflammation [26]. Moreover, we acknowledge FeNO measurements are subject to variation from a number of parameters, including the technique used by the technician, exhalation flow rate and nasal contamination [35]. To minimise the effects of these sampling parameters and ensure consistency across the 10-day sampling period, repeat visits to a child’s home was conducted by the same technician.

With 70 children participating in this study and ten days of consecutive monitoring, a total of 700 observations were possible. A large number of filter samples were, however, excluded from analyses due a number of possible issues including personal sampler flow problems, metal measurement errors or low PM mass loading on filters resulting in metal concentrations below minimum detection limits. The high percentage of data excluded from analyses is an acknowledged caveat of the study, decreasing power and may also have included observations with higher pollutant concentrations. While we were able to observe effects on FeNO, recruitment and trace metal measurement limitations yielded a relatively low sample size especially in stratified analyses examining the modifying effect of medication use and presence of allergies.

## Conclusions

In this panel study of asthmatic children, personal exposure to trace metals on PM_2.5_, representative of traffic and industrial sources, were associated with enhanced FeNO, a biomarker suggested to be indicative of eosinophilic airway inflammation.

## Additional file

Additional file 1: Childhood Asthma and Metals Manuscript SI 2016 08 08R2. (DOCX 187 kb)

## Abbreviations

CI: Confidence interval
FeNO: Forced exhaled nitric oxide
IQR: Interquartile range
PM: Particulate matter
PM_2.5_: Particulate matter with an aerodynamic diameter of 2.5 μm or less
