# Supplementary material for: Trace metal exposure is associated with increased exhaled nitric oxide in asthmatic children
Source: Environ Health. 2016 Sep 1;15(1):94. doi: 10.1186/s12940-016-0173-5 (PMC5009709; doi:10.1186/s12940-016-0173-5)
Supplement: Additional file 1: — Childhood Asthma and Metals Manuscript SI 2016 08 08R2. (DOCX 187 kb) [file 12940_2016_173_MOESM1_ESM.docx]

**Supplemental Information**

TRACE METAL EXPOSURE IS ASSOCIATED WITH INCREASED Exhaled Nitric Oxide in ASTHMATIC Children

**Krystal J. Godri Pollitt,** *Department of Environmental Health Sciences, School of Public Health and Health Sciences, University of Massachusetts, Amherst, Massachusetts, USA*

**Caitlin L. Maikawa,** *Department of Environmental Health Sciences, School of Public Health and Health Sciences, University of Massachusetts, Amherst, Massachusetts, USA*

**Amanda J. Wheeler,** *Health Canada, Air Health Science Division, Ottawa, Ontario, Canada* *and Menzies Institute for Medical Research, University of Tasmania, Private Bag 23, Hobart, Tasmania 7000, Australia*

**Scott Weichenthal,** *Health Canada, Air Health Science Division, Ottawa, Ontario, Canada* and *Department of Epidemiology, Biostatistics, and Occupational Health, McGill University, Montreal, Quebec, Canada*

**Nina A. Dobbin,** *Health Canada, Air Health Science Division, Ottawa, Ontario, Canada*

**Ling Liu,** *Health Canada, Population Studies Division, Ottawa, Ontario, Canada*

**Mark S. Goldberg,** *Department of Medicine, McGill University, Montreal, Canada* and *Division of Clinical Epidemiology, Research Institute, McGill University Health Centre, Montreal, Canada*

**Supplemental Information, Table S1**. Comparison of FeNO of the 70 children in the full and screened data sets for each trace metal assessed. The screened data set was used for all statistical analyses and included trace metal measurements not impacted by personal sampler flow problems, metal measurement errors or low PM_2.5_ mass loading on filters.

|  | **Number of samples** | **FeNO in µL/L, median (*IQR*)** | |
| --- | --- | --- | --- |
| *Full Data Set* | 675 | 17.7 (25.2) | |
|  |  |  | |
| *Screened Data Set* |  |  | |
| Al | 211 | 18.5 (23.5) | |
| As | 86 | 16.7 (17.7) | |
| Ba | 172 | 16.8 (21.5) | |
| Cr | 186 | 18.5 (24.3) | |
| Cu | 210 | 18.4 (22.0) | |
| Fe | 215 | 18.5 (22.0) | |
| Mg | 182 | 16.7 (21.3) | |
| Mn | 207 | 18.5 (22.0) | |
| Ni | 176 | 18.5 (25.0) | |
| S | 217 | 18.5 (22.0) | |
| Sb | 58 | 16.3 (17.3) | |
| Sr | 110 | 24.5 (30.7) | |
| V | 131 | 19.7 (25.5) | |
| Zn | 211 | 18.5 (22.0) | |
|  |  |  |  |

**Supplemental Information, Table S2**. Spearman correlation coefficients of personal PM_2.5_ trace metal exposure concentrations (pg/µg).

|  | **As** | **Ba** | **Cr** | **Cu** | **Fe** | **Mg** | **Mn** | **Ni** | **S** | **Sb** | **Sr** | **V** | **Zn** |
| --- | --- | --- | --- | --- | --- | --- | --- | --- | --- | --- | --- | --- | --- |
| **Al** | 0.38 | 0.49 | 0.16 | 0.48 | 0.84 | 0.47 | 0.71 | 0.55 | 0.72 | 0.33 | 0.14 | 0.52 | 0.62 |
|  | **As** | 0.26 | 0.13 | 0.39 | 0.35 | 0.19 | 0.30 | 0.37 | 0.28 | 0.17 | 0.11 | 0.33 | 0.33 |
|  |  | **Ba** | 0.14 | 0.26 | 0.54 | 0.47 | 0.41 | 0.26 | 0.35 | 0.19 | 0.17 | 0.28 | 0.30 |
|  |  |  | **Cr** | 0.10 | 0.20 | 0.08 | 0.20 | 0.29 | 0.21 | 0.06 | 0.22 | 0.21 | 0.17 |
|  |  |  |  | **Cu** | 0.56 | 0.42 | 0.52 | 0.42 | 0.47 | 0.11 | 0.21 | 0.40 | 0.56 |
|  |  |  |  |  | **Fe** | 0.50 | 0.78 | 0.54 | 0.71 | 0.36 | 0.07 | 0.48 | 0.67 |
|  |  |  |  |  |  | **Mg** | 0.44 | 0.29 | 0.42 | 0.41 | 0.17 | 0.30 | 0.47 |
|  |  |  |  |  |  |  | **Mn** | 0.46 | 0.60 | 0.11 | 0.18 | 0.48 | 0.63 |
|  |  |  |  |  |  |  |  | **Ni** | 0.56 | 0.31 | 0.08 | 0.55 | 0.46 |
|  |  |  |  |  |  |  |  |  | **S** | 0.24 | 0.05 | 0.45 | 0.67 |
|  |  |  |  |  |  |  |  |  |  | **Sb** | 0.03 | 0.28 | 0.28 |
|  |  |  |  |  |  |  |  |  |  |  | **Sr** | 0.09 | 0.14 |
|  |  |  |  |  |  |  |  |  |  |  |  | **V** | 0.35 |
|  | | | | | | | | | | | | | |

**Supplemental Information, Table S3.** Change in the percent change per IQR for metal exposure (pg/µg) with the addition of potential confounding variables to the base, linear mixed model. Results shown are for a 0-day lag exposure period (i.e. the average metal exposure 24 hours prior to the FeNO measurement). The base model included fixed effects for sex and ambient temperature as well as random subject and day of study factors. *The final model included additional personal variables: presence of allergies, eczema before the age of 2, occurrence of an asthma attack in the first year of life, use of beta-agonists, use of corticosteroids and parental asthma. Bolded values indicate personal variables which altered the base model by greater than 10%.*

| ***0-day lag*** | | **% Change per IQR** | **Lower 95% CI** | **Upper 95% CI** | **% Change** | **AIC** |
| --- | --- | --- | --- | --- | --- | --- |
|  |  |  |  |  | **from Base Model** |  |
| **Al** | **Base Model** | 4.1 | -1.2 | 9.7 | - | 221 |
|  | Base Model + **Allergies** (0/1) | 4.2 | -1.1 | 9.8 | 2.1% | 220 |
|  | Base Model + **Asthma Attack in First Year** (0/1) | 4.1 | -1.3 | 9.7 | -1.4% | 221 |
|  | Base Model + **Parental** **Asthma** (0/1) | 4.1 | -1.2 | 9.7 | -0.2% | 222 |
|  | Base Model + **Eczema Before Age 2** (0/1) | 4.1 | -1.2 | 9.7 | -0.3% | 219 |
|  | Base Model + **Beta-agonist Use** (0/1) | 4.1 | -1.2 | 9.8 | 0.4% | 224 |
|  | Base Model + **Corticosteroid Use** (none, irregular, regular) | 4.1 | -1.2 | 9.7 | 0.2% | 222 |
|  | **Final Model** | **4.2** | **-1.1** | **9.7** | **1.0%** | **225** |
|  |  |  |  |  |  |  |
| **As** | **Base Model** | 3.4 | -6.6 | 14.5 | - | 141 |
|  | **Base Model + Allergies** (0/1) | **2.7** | **-7.3** | **13.8** | **-19.8%** | **140** |
|  | **Base Model + Asthma Attack in First Year** (0/1) | **3.0** | **-7.0** | **14.1** | **-10.8%** | **139** |
|  | **Base Model + Parental Asthma** (0/1) | 3.3 | -6.7 | 14.4 | -2.8% | 141 |
|  | **Base Model + Eczema Before Age 2** (0/1) | 3.2 | -6.8 | 14.3 | -7.0% | 140 |
|  | **Base Model + Beta-agonist Use** (0/1) | **4.8** | **-5.7** | **16.6** | **41.5%** | **142** |
|  | **Base Model + Corticosteroid Use** (none, irregular, regular) | 3.3 | -6.8 | 14.4 | -4.1% | 142 |
|  | **Final Model** | **3.5** | **-6.6** | **14.7** | **3.5%** | **141** |
|  |  |  |  |  |  |  |
| **Ba** | **Base Model** | 8.60 | 2.55 | 15.00 | - | 189 |
|  | Base Model + **Allergies** (0/1) | 8.70 | 2.64 | 15.12 | 1.2% | 188 |
|  | Base Model + **Asthma Attack in First Year** (0/1) | 8.67 | 2.61 | 15.08 | 0.8% | 189 |
|  | Base Model + **Parental** **Asthma** (0/1) | 8.59 | 2.54 | 14.99 | -0.2% | 190 |
|  | Base Model + **Eczema Before Age 2** (0/1) | 8.54 | 2.50 | 14.95 | -0.6% | 188 |
|  | Base Model + **Beta-agonist Use** (0/1) | 8.77 | 2.61 | 15.30 | 1.9% | 193 |
|  | Base Model + **Corticosteroid Use** (none, irregular, regular) | 8.63 | 2.58 | 15.04 | 0.4% | 191 |
|  | **Final Model** | **8.93** | **2.81** | **15.41** | **3.8%** | **194** |
|  |  |  |  |  |  |  |
| **Cr** | **Base Model** | 1.83 | -0.29 | 3.99 | - | 205 |
|  | Base Model + **Allergies** (0/1) | 1.82 | -0.30 | 3.98 | -0.5% | 205 |
|  | Base Model + **Asthma Attack in First Year** (0/1) | 1.84 | -0.28 | 4.01 | 0.8% | 205 |
|  | Base Model + **Parental** **Asthma** (0/1) | 1.83 | -0.29 | 3.99 | 0.1% | 206 |
|  | Base Model + **Eczema Before Age 2** (0/1) | 1.82 | -0.30 | 3.98 | -0.5% | 204 |
|  | Base Model + **Beta-agonist Use** (0/1) | 1.83 | -0.31 | 4.02 | 0.4% | 209 |
|  | Base Model + **Corticosteroid Use** (none, irregular, regular) | 1.84 | -0.28 | 4.00 | 0.6% | 207 |
|  | **Final Model** | **1.24** | **-1.41** | **3.97** | **-31.9%** | **225** |

**Supplemental Information, Table S3.** *Continued.*

| ***0-day lag*** | | **% Change per IQR** | **Lower 95% CI** | **Upper 95% CI** | **% Change** | **AIC** |
| --- | --- | --- | --- | --- | --- | --- |
|  |  |  |  |  | **from Base Model** |  |
| **Cu** | **Base Model** | 1.43 | -1.22 | 4.15 | - | 220 |
|  | Base Model + **Allergies** (0/1) | 1.41 | -1.24 | 4.13 | -1.2% | 220 |
|  | Base Model + **Asthma Attack in First Year** (0/1) | 1.33 | -1.33 | 4.05 | -7.3% | 220 |
|  | Base Model + **Parental** **Asthma** (0/1) | 1.39 | -1.27 | 4.11 | -3.1% | 221 |
|  | Base Model + **Eczema Before Age 2** (0/1) | 1.40 | -1.24 | 4.12 | -1.9% | 219 |
|  | Base Model + **Beta-agonist Use** (0/1) | 1.43 | -1.23 | 4.17 | 0.3% | 224 |
|  | Base Model + **Corticosteroid Use** (none, irregular, regular) | 1.42 | -1.23 | 4.14 | -0.5% | 221 |
|  | **Final Model** | **1.84** | **-0.29** | **4.01** | **28.6%** | **209** |
|  |  |  |  |  |  |  |
| **Fe** | **Base Model** | 0.87 | -3.75 | 5.72 | - | 228 |
|  | **Base Model + Allergies** (0/1) | 0.90 | -3.72 | 5.74 | 2.9% | 227 |
|  | **Base Model + Asthma Attack in First Year** (0/1) | **0.77** | **-3.85** | **5.61** | **-11.8%** | **228** |
|  | **Base Model + Parental Asthma** (0/1) | 0.85 | -3.77 | 5.70 | -2.1% | 229 |
|  | **Base Model + Eczema Before Age 2** (0/1) | 0.89 | -3.73 | 5.73 | 1.9% | 227 |
|  | **Base Model + Beta-agonist Use** (0/1) | 0.87 | -3.77 | 5.74 | -0.2% | 232 |
|  | **Base Model + Corticosteroid Use** (none, irregular, regular) | 0.90 | -3.72 | 5.74 | 3.1% | 229 |
|  | **Final Model** | **0.84** | **-3.77** | **5.66** | **-4.1%** | **232** |
|  |  |  |  |  |  |  |
| **Mg** | **Base Model** | 2.20 | -3.39 | 8.12 | - | 216 |
|  | Base Model + **Allergies** (0/1) | 2.29 | -3.31 | 8.22 | 4.4% | 215 |
|  | Base Model + **Asthma Attack in First Year** (0/1) | 2.25 | -3.35 | 8.17 | 2.2% | 215 |
|  | Base Model + **Parental** **Asthma** (0/1) | 2.22 | -3.38 | 8.14 | 0.8% | 216 |
|  | Base Model + **Eczema Before Age 2** (0/1) | 2.18 | -3.42 | 8.09 | -0.9% | 215 |
|  | Base Model + **Beta-agonist Use** (0/1) | 2.17 | -3.45 | 8.11 | -1.4% | 219 |
|  | Base Model + **Corticosteroid Use** (none, irregular, regular) | 2.22 | -3.37 | 8.13 | 0.9% | 217 |
|  | **Final Model** | **2.31** | **-3.27** | **8.21** | **5.1%** | **218** |
|  |  |  |  |  |  |  |
| **Mn** | **Base Model** | -1.19 | -4.66 | 2.42 | - | 214 |
|  | Base Model + **Allergies** (0/1) | -1.15 | -4.63 | 2.46 | -3.1% | 214 |
|  | Base Model + **Asthma Attack in First Year** (0/1) | -1.18 | -4.66 | 2.42 | -0.3% | 214 |
|  | Base Model + **Parental** **Asthma** (0/1) | -1.21 | -4.69 | 2.39 | 2.2% | 215 |
|  | Base Model + **Eczema Before Age 2** (0/1) | -1.20 | -4.68 | 2.40 | 1.4% | 212 |
|  | Base Model + **Beta-agonist Use** (0/1) | -1.22 | -4.71 | 2.40 | 2.7% | 218 |
|  | Base Model + **Corticosteroid Use** (none, irregular, regular) | -1.17 | -4.64 | 2.44 | -1.9% | 215 |
|  | **Final Model** | **-1.20** | **-4.66** | **2.39** | **1.0%** | **217** |
|  |  |  |  |  |  |  |
| **Ni** | **Base Model** | 1.0 | -2.9 | 5.0 | - | 203 |
|  | Base Model + **Allergies** (0/1) | 1.0 | -2.8 | 5.0 | 5.0% | 201 |
|  | Base Model + **Asthma Attack in First Year** (0/1) | 1.0 | -2.9 | 5.0 | -3.3% | 203 |
|  | Base Model + **Parental** **Asthma** (0/1) | 1.0 | -2.9 | 5.0 | -0.7% | 203 |
|  | Base Model + **Eczema Before Age 2** (0/1) | 1.0 | -2.8 | 5.0 | 2.6% | 202 |
|  | Base Model + **Beta-agonist Use** (0/1) | 1.1 | -2.8 | 5.1 | 9.5% | 205 |
|  | Base Model + **Corticosteroid Use** (none, irregular, regular) | 1.0 | -2.8 | 5.0 | 0.9% | 204 |
|  | **Final Model** | **1.15** | **-2.69** | **5.14** | **15.7%** | **206** |

**Supplemental Information, Table S3.** *Continued.*

| ***0-day lag*** | | **% Change per IQR** | **Lower 95% CI** | **Upper 95% CI** | **% Change** | **AIC** |
| --- | --- | --- | --- | --- | --- | --- |
|  |  |  |  |  | **from Base Model** |  |
| **S** | **Base Model** | 2.92 | -1.92 | 8.00 | - | 228 |
|  | Base Model + **Allergies** (0/1) | 2.91 | -1.93 | 7.99 | -0.3% | 228 |
|  | Base Model + **Asthma Attack in First Year** (0/1) | 2.98 | -1.87 | 8.06 | 2.0% | 228 |
|  | Base Model + **Parental** **Asthma** (0/1) | 2.91 | -1.93 | 7.99 | -0.3% | 229 |
|  | Base Model + **Eczema Before Age 2** (0/1) | 3.01 | -1.83 | 8.10 | 3.3% | 227 |
|  | Base Model + **Beta-agonist Use** (0/1) | 2.94 | -1.93 | 8.04 | 0.5% | 232 |
|  | Base Model + **Corticosteroid Use** (none, irregular, regular) | 2.94 | -1.90 | 8.02 | 0.8% | 230 |
|  | **Final Model** | **3.10** | **-1.75** | **8.19** | **6.2%** | **233** |
|  |  |  |  |  |  |  |
| **Sb** | **Base Model** | -0.91 | -15.20 | 15.80 | - | 127 |
|  | **Base Model + Allergies (0/1)** | **-0.16** | **-14.64** | **16.77** | **-81.9%** | **127** |
|  | **Base Model + Asthma Attack in First Year (0/1)** | **-0.80** | **-15.14** | **15.96** | **-11.7%** | **128** |
|  | **Base Model + Parental Asthma (0/1)** | -0.90 | -15.26 | 15.90 | -0.7% | 128 |
|  | **Base Model + Eczema Before Age 2 (0/1)** | **-1.00** | **-15.29** | **15.70** | **10.4%** | **127** |
|  | **Base Model + Beta-agonist Use (0/1)** | **-0.23** | **-17.18** | **20.19** | **-74.8%** | **129** |
|  | **Base Model + Corticosteroid Use** (none, irregular, regular) | -0.82 | -15.16 | 15.93 | -9.3% | 129 |
|  | **Final Model** | **1.75** | **-14.69** | **21.34** | **-292.5%** | **130** |
|  |  |  |  |  |  |  |
| **Sr** | **Base Model** | 1.68 | -3.23 | 6.85 | - | 162 |
|  | Base Model + **Allergies** (0/1) | 1.69 | -3.24 | 6.87 | 0.3% | 162 |
|  | Base Model + **Asthma Attack in First Year** (0/1) | 1.62 | -3.30 | 6.80 | -3.5% | 161 |
|  | Base Model + **Parental** **Asthma** (0/1) | 1.64 | -3.27 | 6.80 | -2.6% | 163 |
|  | Base Model + **Eczema Before Age 2** (0/1) | 1.55 | -3.37 | 6.72 | -7.8% | 161 |
|  | Base Model + **Beta-agonist Use** (0/1) | 1.70 | -3.31 | 6.97 | 1.2% | 165 |
|  | Base Model + **Corticosteroid Use** (none, irregular, regular) | 1.70 | -3.23 | 6.88 | 0.9% | 164 |
|  | **Final Model** | **1.41** | **-3.53** | **6.61** | **-16.3%** | **165** |
|  |  |  |  |  |  |  |
| **V** | **Base Model** | 4.6 | -3.3 | 13.2 | - | 163 |
|  | Base Model + **Allergies** (0/1) | 4.7 | -3.2 | 13.3 | 3.3% | 160 |
|  | Base Model + **Asthma Attack in First Year** (0/1) | 4.6 | -3.3 | 13.2 | -0.1% | 164 |
|  | Base Model + **Parental** **Asthma** (0/1) | 4.7 | -3.2 | 13.3 | 2.0% | 163 |
|  | Base Model + **Eczema Before Age 2** (0/1) | 4.6 | -3.3 | 13.1 | -0.5% | 163 |
|  | Base Model + **Beta-agonist Use** (0/1) | 4.8 | -3.2 | 13.5 | 4.5% | 166 |
|  | Base Model + **Corticosteroid Use** (none, irregular, regular) | 4.6 | -3.3 | 13.1 | -0.2% | 164 |
|  | **Final Model** | **4.97** | **-2.94** | **13.53** | **8.2%** | **166** |
|  |  |  |  |  |  |  |
| **Zn** | **Base Model** | -1.0 | -4.3 | 2.5 | - | 217 |
|  | Base Model + **Allergies** (0/1) | -1.0 | -4.4 | 2.4 | 6.5% | 216 |
|  | Base Model + **Asthma Attack in First Year** (0/1) | -1.0 | -4.3 | 2.5 | 1.5% | 217 |
|  | Base Model + **Parental** **Asthma** (0/1) | -1.0 | -4.3 | 2.5 | 2.9% | 218 |
|  | Base Model + **Eczema Before Age 2** (0/1) | -0.9 | -4.2 | 2.6 | -6.9% | 217 |
|  | Base Model + **Beta-agonist Use** (0/1) | -1.0 | -4.3 | 2.5 | -0.2% | 221 |
|  | Base Model + **Corticosteroid Use** (none, irregular, regular) | -0.9 | -4.2 | 2.5 | -5.4% | 218 |
|  | **Final Model** | **-0.93** | **-4.26** | **2.51** | **-2.9%** | **222** |

**Supplemental Information, Table S4.** Change in the percent change per IQR for metal exposure (pg/µg) with the addition of potential confounding variables to the base, linear mixed model. Results shown are for a 1-day lag exposure period (i.e. the average metal exposure 24-48 hours prior to the FeNO measurement). The base model included fixed effects for sex and ambient temperature as well as random subject and day of study factors. *The final model included additional personal variables: presence of allergies, eczema before the age of 2, occurrence of an asthma attack in the first year of life, use of beta-agonists, use of corticosteroids and parental asthma.* *Bolded values indicate personal variables which altered the base model by greater than 10%.*

| ***1-day lag*** | | **% Change per IQR** | **Lower 95% CI** | **Upper 95% CI** | **% Change** | **AIC** |
| --- | --- | --- | --- | --- | --- | --- |
|  |  |  |  |  | **from Base Model** |  |
| **Al** | **Base Model** | 10.0 | 3.9 | 16.5 | - | 198 |
|  | Base Model + **Allergies** (0/1) | 10.2 | 4.1 | 16.7 | 5.9% | 196 |
|  | Base Model + **Asthma Attack in First Year** (0/1) | 10.0 | 3.9 | 16.5 | -0.5% | 199 |
|  | Base Model + **Parental** **Asthma** (0/1) | 10.0 | 3.9 | 16.5 | -0.1% | 199 |
|  | Base Model + **Eczema Before Age 2** (0/1) | 10.0 | 3.9 | 16.5 | -0.2% | 198 |
|  | Base Model + **Beta-agonist Use** (0/1) | 10.2 | 4.1 | 16.6 | 1.5% | 200 |
|  | Base Model + **Corticosteroid Use** (none, irregular, regular) | 10.0 | 3.9 | 16.5 | 0.2% | 200 |
|  | **Final Model** | **10.3** | **4.2** | **16.6** | **2.3%** | **203** |
|  |  |  |  |  |  |  |
| **As** | **Base Model** | 9.0 | -1.5 | 20.7 | - | 111 |
|  | **Base Model + Allergies** (0/1) | 8.4 | -2.0 | 20.0 | -6.5% | 110 |
|  | **Base Model + Asthma Attack in First Year** (0/1) | 8.6 | -1.9 | 20.3 | -4.4% | 111 |
|  | **Base Model + Parental Asthma** (0/1) | 9.1 | -1.5 | 20.8 | 1.0% | 112 |
|  | **Base Model + Eczema Before Age 2** (0/1) | 9.0 | -1.6 | 20.6 | -0.6% | 112 |
|  | **Base Model + Beta-agonist Use** (0/1) | 9.2 | -1.9 | 21.6 | 2.2% | 114 |
|  | **Base Model + Corticosteroid Use** (none, irregular, regular) | 9.0 | -1.5 | 20.7 | 0.3% | 113 |
|  | **Final Model** | **8.4** | **-2.4** | **20.3** | **-7.0%** | **115** |
|  |  |  |  |  |  |  |
| **Ba** | **Base Model** | 3.5 | -2.8 | 10.2 | - | 194 |
|  | Base Model + **Allergies** (0/1) | 3.7 | -2.6 | 10.4 | 5.0% | 193 |
|  | Base Model + **Asthma Attack in First Year** (0/1) | 3.5 | -2.8 | 10.3 | 1.0% | 195 |
|  | Base Model + **Parental** **Asthma** (0/1) | 3.5 | -2.8 | 10.3 | 1.3% | 195 |
|  | Base Model + **Eczema Before Age 2** (0/1) | 3.5 | -2.8 | 10.2 | -1.2% | 194 |
|  | Base Model + **Beta-agonist Use** (0/1) | **2.9** | **-3.4** | **9.6** | **-16.9%** | **196** |
|  | Base Model + **Corticosteroid Use** (none, irregular, regular) | 3.5 | -2.8 | 10.2 | 0.6% | 196 |
|  | **Final Model** | **3.2** | **-3.1** | **9.8** | **-9.9%** | **198** |
|  |  |  |  |  |  |  |
| **Cr** | **Base Model** | 0.4 | -1.8 | 2.7 | - | 196 |
|  | Base Model + **Allergies** (0/1) | 0.4 | -1.8 | 2.7 | -3.5% | 195 |
|  | Base Model + **Asthma Attack in First Year** (0/1) | 0.4 | -1.8 | 2.7 | 2.8% | 197 |
|  | Base Model + **Parental** **Asthma** (0/1) | 0.4 | -1.8 | 2.7 | -1.4% | 197 |
|  | Base Model + **Eczema Before Age 2** (0/1) | 0.4 | -1.8 | 2.7 | -1.0% | 197 |
|  | Base Model + **Beta-agonist Use** (0/1) | **0.4** | **-1.8** | **2.6** | **-17.6%** | **199** |
|  | Base Model + **Corticosteroid Use** (none, irregular, regular) | 0.4 | -1.8 | 2.7 | -0.3% | 198 |
|  | **Final Model** | **2.6** | **-1.0** | **6.2** | **497.2%** | **205** |

**Supplemental Information, Table S4.** *Continued.*

| ***1-day lag*** | | **% Change per IQR** | **Lower 95% CI** | **Upper 95% CI** | **% Change** | **AIC** |
| --- | --- | --- | --- | --- | --- | --- |
|  |  |  |  |  | **from Base Model** |  |
| **Cu** | **Base Model** | 2.8 | -0.8 | 6.4 | - | 199 |
|  | Base Model + **Allergies** (0/1) | 2.7 | -0.8 | 6.4 | -0.6% | 198 |
|  | Base Model + **Asthma Attack in First Year** (0/1) | 2.7 | -0.9 | 6.3 | -3.4% | 200 |
|  | Base Model + **Parental** **Asthma** (0/1) | 2.7 | -0.8 | 6.4 | -1.0% | 201 |
|  | Base Model + **Eczema Before Age 2** (0/1) | 2.7 | -0.8 | 6.4 | -1.0% | 200 |
|  | Base Model + **Beta-agonist Use** (0/1) | 2.7 | -0.8 | 6.4 | -0.5% | 202 |
|  | Base Model + **Corticosteroid Use** (none, irregular, regular) | 2.7 | -0.8 | 6.4 | -1.3% | 201 |
|  | **Final Model** | **0.3** | **-1.8** | **2.6** | **-87.8%** | **202** |
|  |  |  |  |  |  |  |
| **Fe** | **Base Model** | 7.2 | 1.2 | 13.6 | - | 196 |
|  | **Base Model + Allergies** (0/1) | 7.3 | 1.2 | 13.7 | 1.2% | 194 |
|  | **Base Model + Asthma Attack in First Year** (0/1) | 7.2 | 1.1 | 13.6 | -0.2% | 197 |
|  | **Base Model + Parental Asthma** (0/1) | 7.2 | 1.2 | 13.6 | 0.0% | 197 |
|  | **Base Model + Eczema Before Age 2** (0/1) | 7.2 | 1.2 | 13.6 | 0.6% | 196 |
|  | **Base Model + Beta-agonist Use** (0/1) | 7.3 | 1.3 | 13.7 | 1.5% | 198 |
|  | **Base Model + Corticosteroid Use** (none, irregular, regular) | 7.3 | 1.2 | 13.7 | 1.2% | 198 |
|  | **Final Model** | **7.5** | **1.5** | **13.9** | **4.6%** | **201** |
|  |  |  |  |  |  |  |
| **Mg** | **Base Model** | 7.6 | 1.7 | 13.9 | - | 171 |
|  | Base Model + **Allergies** (0/1) | 7.8 | 1.9 | 14.1 | 2.2% | 169 |
|  | Base Model + **Asthma Attack in First Year** (0/1) | 7.7 | 1.7 | 13.9 | 0.6% | 172 |
|  | Base Model + **Parental** **Asthma** (0/1) | 7.6 | 1.7 | 13.9 | 0.1% | 172 |
|  | Base Model + **Eczema Before Age 2** (0/1) | 7.6 | 1.7 | 13.8 | -0.6% | 171 |
|  | Base Model + **Beta-agonist Use** (0/1) | 7.5 | 1.6 | 13.8 | -1.3% | 175 |
|  | Base Model + **Corticosteroid Use** (none, irregular, regular) | 7.7 | 1.7 | 13.9 | 0.5% | 173 |
|  | **Final Model** | **7.7** | **1.8** | **13.9** | **1.1%** | **175** |
|  |  |  |  |  |  |  |
| **Mn** | **Base Model** | -0.8 | -5.0 | 3.6 | - | 196 |
|  | Base Model + **Allergies** (0/1) | -0.7 | -4.9 | 3.7 | -9.5% | 195 |
|  | Base Model + **Asthma Attack in First Year** (0/1) | -0.8 | -5.0 | 3.6 | -5.2% | 197 |
|  | Base Model + **Parental** **Asthma** (0/1) | -0.8 | -5.0 | 3.6 | 3.4% | 197 |
|  | Base Model + **Eczema Before Age 2** (0/1) | -0.8 | -5.0 | 3.6 | -1.9% | 195 |
|  | Base Model + **Beta-agonist Use** (0/1) | **-0.7** | **-4.9** | **3.7** | **-17.1%** | **199** |
|  | Base Model + **Corticosteroid Use** (none, irregular, regular) | -0.8 | -5.0 | 3.6 | -5.5% | 198 |
|  | **Final Model** | **-0.5** | **-4.7** | **3.9** | **-34.7%** | **200** |
|  |  |  |  |  |  |  |
| **Ni** | **Base Model** | 1.2 | -3.4 | 6.0 | - | 191 |
|  | Base Model + **Allergies** (0/1) | 1.2 | -3.4 | 6.0 | 3.2% | 189 |
|  | Base Model + **Asthma Attack in First Year** (0/1) | 1.1 | -3.4 | 5.9 | -1.1% | 192 |
|  | Base Model + **Parental** **Asthma** (0/1) | 1.1 | -3.4 | 5.9 | -1.7% | 192 |
|  | Base Model + **Eczema Before Age 2** (0/1) | 1.2 | -3.4 | 6.0 | 5.3% | 190 |
|  | Base Model + **Beta-agonist Use** (0/1) | **1.5** | **-2.9** | **6.0** | **26.1%** | **189** |
|  | Base Model + **Corticosteroid Use** (none, irregular, regular) | 1.2 | -3.4 | 6.0 | 1.2% | 192 |
|  | **Final Model** | **1.5** | **-2.8** | **6.1** | **33.7%** | **190** |

**Supplemental Information, Table S4.** *Continued.*

| ***1-day lag*** | | **% Change per IQR** | **Lower 95% CI** | **Upper 95% CI** | **% Change** | **AIC** |
| --- | --- | --- | --- | --- | --- | --- |
|  |  |  |  |  | **from Base Model** |  |
| **S** | **Base Model** | 2.4 | -3.0 | 8.2 | - | 207 |
|  | Base Model + **Allergies** (0/1) | 2.5 | -3.0 | 8.2 | 0.9% | 206 |
|  | Base Model + **Asthma Attack in First Year** (0/1) | 2.4 | -3.0 | 8.2 | 0.4% | 208 |
|  | Base Model + **Parental** **Asthma** (0/1) | **2.4** | **-3.0** | **8.2** | **-17.2%** | **208** |
|  | Base Model + **Eczema Before Age 2** (0/1) | 2.5 | -3.0 | 8.2 | 1.4% | 207 |
|  | Base Model + **Beta-agonist Use** (0/1) | 2.4 | -3.1 | 8.2 | -1.3% | 210 |
|  | Base Model + **Corticosteroid Use** (none, irregular, regular) | **2.5** | **-3.0** | **8.2** | **-15.6%** | **209** |
|  | **Final Model** | **2.5** | **-2.9** | **8.2** | **2.4%** | **213** |
|  |  |  |  |  |  |  |
| **Sb** | **Base Model** | False Convergence | | | | |
|  | **Base Model + Allergies** (0/1) | False Convergence | | | | |
|  | **Base Model + Asthma Attack in First Year** (0/1) | False Convergence | | | | |
|  | **Base Model + Parental Asthma** (0/1) | False Convergence | | | | |
|  | **Base Model + Eczema Before Age 2** (0/1) | False Convergence | | | | |
|  | **Base Model + Beta-agonist Use** (0/1) | False Convergence | | | | |
|  | **Base Model + Corticosteroid Use** (none, irregular, regular) | False Convergence | | | | |
|  | **Final Model** | **False Convergence** | | | | |
|  |  |  |  |  |  |  |
| **Sr** | **Base Model** | -0.8 | -5.8 | 4.6 | - | 138 |
|  | Base Model + **Allergies** (0/1) | -0.8 | -5.8 | 4.6 | -0.6% | 137 |
|  | Base Model + **Asthma Attack in First Year** (0/1) | -0.8 | -5.9 | 4.5 | 5.3% | 138 |
|  | Base Model + **Parental** **Asthma** (0/1) | **-0.8** | **-5.8** | **4.5** | **-146.7%** | **139** |
|  | Base Model + **Eczema Before Age 2** (0/1) | -0.8 | -5.9 | 4.5 | 7.7% | 138 |
|  | Base Model + **Beta-agonist Use** (0/1) | **-1.4** | **-6.1** | **3.6** | **81.3%** | **139** |
|  | Base Model + **Corticosteroid Use** (none, irregular, regular) | **-0.7** | **-5.8** | **4.6** | **-143.2%** | **140** |
|  | **Final Model** | **-1.5** | **-6.2** | **3.4** | **97.8%** | **140** |
|  |  |  |  |  |  |  |
| **V** | **Base Model** | 0.4 | -6.4 | 7.8 | - | 168 |
|  | Base Model + **Allergies** (0/1) | **0.7** | **-6.2** | **8.2** | **66.6%** | **166** |
|  | Base Model + **Asthma Attack in First Year** (0/1) | 0.4 | -6.5 | 7.8 | -8.5% | 168 |
|  | Base Model + **Parental** **Asthma** (0/1) | 0.5 | -6.4 | 7.9 | 1.8% | 169 |
|  | Base Model + **Eczema Before Age 2** (0/1) | **0.4** | **-6.5** | **7.8** | **-12.2%** | **167** |
|  | Base Model + **Beta-agonist Use** (0/1) | **0.6** | **-6.4** | **8.0** | **26.4%** | **171** |
|  | Base Model + **Corticosteroid Use** (none, irregular, regular) | **0.5** | **-6.4** | **7.9** | **10.7%** | **169** |
|  | **Final Model** | **0.8** | **-6.1** | **8.1** | **72.1%** | **171** |
|  |  |  |  |  |  |  |
| **Zn** | **Base Model** | 1.6 | -1.8 | 5.1 | - | 202 |
|  | Base Model + **Allergies** (0/1) | 1.5 | -1.9 | 5.1 | -5.4% | 201 |
|  | Base Model + **Asthma Attack in First Year** (0/1) | 1.6 | -1.8 | 5.1 | -0.6% | 203 |
|  | Base Model + **Parental** **Asthma** (0/1) | 1.6 | -1.8 | 5.1 | -1.2% | 203 |
|  | Base Model + **Eczema Before Age 2** (0/1) | 1.7 | -1.8 | 5.2 | 4.0% | 202 |
|  | Base Model + **Beta-agonist Use** (0/1) | 1.7 | -1.8 | 5.2 | 3.1% | 205 |
|  | Base Model + **Corticosteroid Use** (none, irregular, regular) | 1.6 | -1.8 | 5.2 | 1.7% | 204 |
|  | **Final Model** | **1.7** | **-1.8** | **5.2** | **2.9%** | **208** |

**Supplemental Information, Table S5.** Change in the percent change per IQR for metal exposure (pg/µg) with the addition of potential confounding variables to the base, linear mixed model. Results shown are for a 2-day lag exposure period (i.e. the average metal exposure 48-72 hours prior to the FeNO measurement). The base model included fixed effects for sex and ambient temperature as well as random subject and day of study factors. *The final model included additional personal variables: presence of allergies, eczema before the age of 2, occurrence of an asthma attack in the first year of life, use of beta-agonists, use of corticosteroids and parental asthma.* *Bolded values indicate personal variables which altered the base model by greater than 10%.*

| ***2-day lag*** | | **% Change per IQR** | **Lower 95% CI** | **Upper 95% CI** | **% Change** | **AIC** |
| --- | --- | --- | --- | --- | --- | --- |
|  |  |  |  |  | **from Base Model** |  |
| **Al** | **Base Model** | 6.3 | 0.0 | 12.9 | - | 192 |
|  | Base Model + **Allergies** (0/1) | **6.3** | **0.0** | **13.0** | **36.6%** | **191** |
|  | Base Model + **Asthma Attack in First Year** (0/1) | 6.2 | 0.0 | 12.9 | -0.7% | 192 |
|  | Base Model + **Parental** **Asthma** (0/1) | 6.3 | 0.0 | 12.9 | 0.0% | 192 |
|  | Base Model + **Eczema Before Age 2** (0/1) | 6.2 | 0.0 | 12.9 | -0.5% | 190 |
|  | Base Model + **Beta-agonist Use** (0/1) | 6.0 | -0.1 | 12.6 | -3.4% | 192 |
|  | Base Model + **Corticosteroid Use** (none, irregular, regular) | 6.3 | 0.0 | 13.0 | 0.5% | 193 |
|  | **Final Model** | **6.0** | **0.0** | **12.5** | **-3.6%** | **191** |
|  |  |  |  |  |  |  |
| **As** | **Base Model** | 1.4 | -9.8 | 14.0 | - | 108 |
|  | **Base Model + Allergies** (0/1) | **0.9** | **-10.2** | **13.4** | **-32.8%** | **107** |
|  | **Base Model + Asthma Attack in First Year** (0/1) | **1.1** | **-10.1** | **13.6** | **-21.8%** | **107** |
|  | **Base Model + Parental Asthma** (0/1) | 1.5 | -9.7 | 14.1 | 9.8% | 109 |
|  | **Base Model + Eczema Before Age 2** (0/1) | **1.0** | **-10.1** | **13.4** | **-29.8%** | **107** |
|  | **Base Model + Beta-agonist Use** (0/1) | **5.0** | **-6.9** | **18.4** | **263.9%** | **109** |
|  | **Base Model + Corticosteroid Use** (none, irregular, regular) | 1.5 | -9.7 | 14.1 | 8.1% | 110 |
|  | **Final Model** | **4.3** | **-6.9** | **16.8** | **210.4%** | **107** |
|  |  |  |  |  |  |  |
| **Ba** | **Base Model** | 5.20 | -2.23 | 13.19 | - | 179 |
|  | Base Model + **Allergies** (0/1) | 5.33 | -2.11 | 13.34 | 2.6% | 179 |
|  | Base Model + **Asthma Attack in First Year** (0/1) | 5.23 | -2.21 | 13.22 | 0.6% | 180 |
|  | Base Model + **Parental** **Asthma** (0/1) | 5.14 | -2.30 | 13.14 | -1.1% | 180 |
|  | Base Model + **Eczema Before Age 2** (0/1) | 5.21 | -2.22 | 13.20 | 0.2% | 178 |
|  | Base Model + **Beta-agonist Use** (0/1) | **4.48** | **-2.99** | **12.52** | **-13.9%** | **182** |
|  | Base Model + **Corticosteroid Use** (none, irregular, regular) | 5.21 | -2.22 | 13.21 | 0.3% | 181 |
|  | **Final Model** | **4.56** | **-2.82** | **12.51** | **-12.2%** | **183** |
|  |  |  |  |  |  |  |
| **Cr** | **Base Model** | 0.73 | -1.24 | 2.74 | - | 168 |
|  | Base Model + **Allergies** (0/1) | 0.72 | -1.25 | 2.72 | -2.1% | 167 |
|  | Base Model + **Asthma Attack in First Year** (0/1) | 0.74 | -1.23 | 2.75 | 1.4% | 167 |
|  | Base Model + **Parental** **Asthma** (0/1) | 0.73 | -1.24 | 2.74 | -0.1% | 169 |
|  | Base Model + **Eczema Before Age 2** (0/1) | 0.73 | -1.23 | 2.74 | 0.3% | 167 |
|  | Base Model + **Beta-agonist Use** (0/1) | 0.70 | -1.27 | 2.70 | -4.6% | 171 |
|  | Base Model + **Corticosteroid Use** (none, irregular, regular) | 0.74 | -1.22 | 2.75 | 1.8% | 170 |
|  | **Final Model** | **0.13** | **-3.54** | **3.94** | **-82.5%** | **195** |

**Supplemental Information, Table S5.** *Continued.*

| ***2-day lag*** | | **% Change per IQR** | **Lower 95% CI** | **Upper 95% CI** | **% Change** | **AIC** |
| --- | --- | --- | --- | --- | --- | --- |
|  |  |  |  |  | **from Base Model** |  |
| **Cu** | **Base Model** | 1.04 | -2.71 | 4.93 | - | 195 |
|  | Base Model + **Allergies** (0/1) | 1.02 | -2.72 | 4.90 | -1.7% | 194 |
|  | Base Model + **Asthma Attack in First Year** (0/1) | **0.93** | **-2.82** | **4.82** | **-10.5%** | **195** |
|  | Base Model + **Parental** **Asthma** (0/1) | 0.96 | -2.79 | 4.86 | -6.9% | 196 |
|  | Base Model + **Eczema Before Age 2** (0/1) | 0.95 | -2.78 | 4.83 | -8.0% | 193 |
|  | Base Model + **Beta-agonist Use** (0/1) | **0.48** | **-3.24** | **4.34** | **-53.7%** | **195** |
|  | Base Model + **Corticosteroid Use** (none, irregular, regular) | 1.01 | -2.74 | 4.90 | -2.4% | 197 |
|  | **Final Model** | **0.71** | **-1.23** | **2.68** | **-31.7%** | **171** |
|  |  |  |  |  |  |  |
| **Fe** | **Base Model** | 3.76 | -2.28 | 10.17 | - | 192 |
|  | **Base Model + Allergies** (0/1) | 3.78 | -2.26 | 10.19 | 0.5% | 192 |
|  | **Base Model + Asthma Attack in First Year** (0/1) | 3.73 | -2.31 | 10.14 | -0.8% | 193 |
|  | **Base Model + Parental Asthma** (0/1) | 3.72 | -2.32 | 10.14 | -0.9% | 193 |
|  | **Base Model + Eczema Before Age 2** (0/1) | 3.85 | -2.19 | 10.26 | 2.4% | 191 |
|  | **Base Model + Beta-agonist Use** (0/1) | **2.84** | **-3.11** | **9.15** | **-24.5%** | **193** |
|  | **Base Model + Corticosteroid Use** (none, irregular, regular) | 3.87 | -2.19 | 10.30 | 3.0% | 194 |
|  | **Final Model** | **2.97** | **-2.92** | **9.22** | **-19.2%** | **193** |
|  |  |  |  |  |  |  |
| **Mg** | **Base Model** | 5.67 | -0.61 | 12.34 | - | 181 |
|  | Base Model + **Allergies** (0/1) | 5.75 | -0.53 | 12.42 | 1.3% | 180 |
|  | Base Model + **Asthma Attack in First Year** (0/1) | 5.72 | -0.56 | 12.40 | 0.9% | 181 |
|  | Base Model + **Parental** **Asthma** (0/1) | 5.70 | -0.58 | 12.38 | 0.6% | 181 |
|  | Base Model + **Eczema Before Age 2** (0/1) | 5.55 | -0.71 | 12.22 | -2.1% | 180 |
|  | Base Model + **Beta-agonist Use** (0/1) | 5.58 | -0.57 | 12.11 | -1.6% | 182 |
|  | Base Model + **Corticosteroid Use** (none, irregular, regular) | 5.67 | -0.60 | 12.35 | 0.1% | 183 |
|  | **Final Model** | **5.57** | **-0.50** | **12.01** | **-1.7%** | **182** |
|  |  |  |  |  |  |  |
| **Mn** | **Base Model** | 2.05 | -3.13 | 7.50 | - | 180 |
|  | Base Model + **Allergies** (0/1) | 2.17 | -3.01 | 7.63 | 6.1% | 179 |
|  | Base Model + **Asthma Attack in First Year** (0/1) | 2.18 | -3.00 | 7.65 | 6.5% | 180 |
|  | Base Model + **Parental** **Asthma** (0/1) | 1.96 | -3.23 | 7.42 | -4.3% | 181 |
|  | Base Model + **Eczema Before Age 2** (0/1) | 1.99 | -3.16 | 7.42 | -2.9% | 176 |
|  | Base Model + **Beta-agonist Use** (0/1) | **2.28** | **-2.83** | **7.67** | **11.5%** | **180** |
|  | Base Model + **Corticosteroid Use** (none, irregular, regular) | 2.09 | -3.10 | 7.54 | 1.8% | 181 |
|  | **Final Model** | **2.36** | **-2.70** | **7.68** | **15.2%** | **178** |
|  |  |  |  |  |  |  |
| **Ni** | **Base Model** | 2.13 | -2.18 | 6.64 | - | 179 |
|  | Base Model + **Allergies** (0/1) | 2.15 | -2.17 | 6.66 | 0.8% | 178 |
|  | Base Model + **Asthma Attack in First Year** (0/1) | 2.17 | -2.15 | 6.68 | 1.6% | 180 |
|  | Base Model + **Parental** **Asthma** (0/1) | 2.14 | -2.18 | 6.65 | 0.5% | 180 |
|  | Base Model + **Eczema Before Age 2** (0/1) | 2.20 | -2.12 | 6.71 | 3.0% | 178 |
|  | Base Model + **Beta-agonist Use** (0/1) | 2.20 | -2.11 | 6.71 | 3.2% | 182 |
|  | Base Model + **Corticosteroid Use** (none, irregular, regular) | 2.15 | -2.17 | 6.66 | 0.6% | 181 |
|  | **Final Model** | **2.37** | **-1.90** | **6.81** | **10.9%** | **183** |

**Supplemental Information, Table S5.** *Continued.*

| ***2-day lag*** | | **% Change per IQR** | **Lower 95% CI** | **Upper 95% CI** | **% Change** | **AIC** |
| --- | --- | --- | --- | --- | --- | --- |
|  |  |  |  |  | **from Base Model** |  |
| **S** | **Base Model** | 4.01 | -1.64 | 9.98 | - | 195 |
|  | Base Model + **Allergies** (0/1) | 3.95 | -1.69 | 9.92 | -1.4% | 194 |
|  | Base Model + **Asthma Attack in First Year** (0/1) | 4.01 | -1.64 | 9.98 | 0.0% | 195 |
|  | Base Model + **Parental** **Asthma** (0/1) | **4.00** | **-1.64** | **9.97** | **36.9%** | **196** |
|  | Base Model + **Eczema Before Age 2** (0/1) | 4.01 | -1.63 | 9.97 | 0.0% | 193 |
|  | Base Model + **Beta-agonist Use** (0/1) | **3.38** | **-2.20** | **9.28** | **-15.6%** | **195** |
|  | Base Model + **Corticosteroid Use** (none, irregular, regular) | **4.01** | **-1.64** | **9.98** | **37.4%** | **197** |
|  | **Final Model** | **3.30** | **-2.21** | **9.12** | **-17.7%** | **195** |
|  |  |  |  |  |  |  |
| **Sb** | **Base Model** | 2.14 | -11.76 | 18.23 | - | 100 |
|  | **Base Model + Allergies (0/1)** | **2.76** | **-11.21** | **18.93** | **29.0%** | **98** |
|  | **Base Model + Asthma Attack in First Year (0/1)** | 2.30 | -11.66 | 18.45 | 7.4% | 100 |
|  | **Base Model + Parental Asthma (0/1)** | 2.24 | -11.70 | 18.38 | 4.6% | 100 |
|  | **Base Model + Eczema Before Age 2 (0/1)** | 2.25 | -11.67 | 18.37 | 5.5% | 99 |
|  | **Base Model + Beta-agonist Use (0/1)** | **1.26** | **-15.36** | **21.15** | **-40.9%** | **101** |
|  | **Base Model + Corticosteroid Use** (none, irregular, regular) | 2.05 | -11.92 | 18.24 | -4.1% | 101 |
|  | **Final Model** | **2.69** | **-12.78** | **20.90** | **25.8%** | **102** |
|  |  |  |  |  |  |  |
| **Sr** | **Base Model** | 1.5 | -1.7 | 4.8 | - | 119 |
|  | Base Model + **Allergies** (0/1) | 1.5 | -1.6 | 4.8 | -0.2% | 119 |
|  | Base Model + **Asthma Attack in First Year** (0/1) | 1.4 | -1.7 | 4.7 | -5.1% | 117 |
|  | Base Model + **Parental** **Asthma** (0/1) | **1.5** | **-1.7** | **4.8** | **-12.1%** | **119** |
|  | Base Model + **Eczema Before Age 2** (0/1) | 1.5 | -1.7 | 4.8 | -1.5% | 118 |
|  | Base Model + **Beta-agonist Use** (0/1) | 1.5 | -1.7 | 4.9 | 0.6% | 122 |
|  | Base Model + **Corticosteroid Use** (none, irregular, regular) | 1.5 | -1.6 | 4.8 | -9.8% | 121 |
|  | **Final Model** | **1.38** | **-1.75** | **4.62** | **-8.5%** | **120** |
|  |  |  |  |  |  |  |
| **V** | **Base Model** | 7.15 | -0.42 | 15.28 | - | 163 |
|  | Base Model + **Allergies** (0/1) | 7.30 | -0.28 | 15.45 | 2.2% | 162 |
|  | Base Model + **Asthma Attack in First Year** (0/1) | 7.18 | -0.38 | 15.31 | 0.5% | 163 |
|  | Base Model + **Parental** **Asthma** (0/1) | 7.27 | -0.31 | 15.41 | 1.7% | 163 |
|  | Base Model + **Eczema Before Age 2** (0/1) | 7.10 | -0.45 | 15.22 | -0.6% | 162 |
|  | Base Model + **Beta-agonist Use** (0/1) | 7.33 | -0.36 | 15.61 | 2.6% | 166 |
|  | Base Model + **Corticosteroid Use** (none, irregular, regular) | 7.17 | -0.40 | 15.32 | 0.3% | 165 |
|  | **Final Model** | **7.64** | **0.08** | **15.77** | **6.9%** | **166** |
|  |  |  |  |  |  |  |
| **Zn** | **Base Model** | 2.46 | -1.34 | 6.41 | - | 192 |
|  | Base Model + **Allergies** (0/1) | 2.35 | -1.45 | 6.30 | -4.6% | 191 |
|  | Base Model + **Asthma Attack in First Year** (0/1) | 2.44 | -1.37 | 6.38 | -1.0% | 192 |
|  | Base Model + **Parental** **Asthma** (0/1) | 2.42 | -1.38 | 6.37 | -1.5% | 193 |
|  | Base Model + **Eczema Before Age 2** (0/1) | **-0.98** | **-4.31** | **2.48** | **-139.7%** | **217** |
|  | Base Model + **Beta-agonist Use** (0/1) | **1.87** | **-1.90** | **5.79** | **-23.8%** | **192** |
|  | Base Model + **Corticosteroid Use** (none, irregular, regular) | 2.52 | -1.29 | 6.48 | 2.4% | 194 |
|  | **Final Model** | **1.88** | **-1.85** | **5.75** | **-23.6%** | **192** |

**Supplemental Information, Table S6.** Percent change in FeNO per IQR change (95% confidence limit) in metal concentration (pg/µg). The mixed models included a random effect for each child, a first-order autoregressive correlation structure, and indicators for day (1-10) of the study. Models were adjusted for fixed effects including temperature, sex, the presence of allergies, eczema before the age of 2, occurrence of an asthma attack in previous 12 months, use of beta-agonists, use of corticosteroids and parental asthma.

| **Exposure** | **Exposure** | **% Change** | **Lower** | **Upper** | **Total Number of** |
| --- | --- | --- | --- | --- | --- |
| **Metric** | **Lag Period** | **per IQR** | **95% CI** | **95% CI** | **Observations** |
| **Al** | 0-day | 4.2 | -1.1 | 9.7 | 208 |
|  | 1-day | 10.3 | 4.2 | 16.6 | 188 |
|  | 2-day | 6.0 | 0.0 | 12.5 | 169 |
|  |  |  |  |  |  |
| **As** | 0-day | 3.5 | -6.6 | 14.7 | 84 |
|  | 1-day | 8.4 | -2.4 | 20.3 | 73 |
|  | 2-day | 4.3 | -6.9 | 16.8 | 63 |
|  |  |  |  |  |  |
| **Ba** | 0-day | 8.9 | 2.8 | 15.4 | 169 |
|  | 1-day | 3.2 | -3.1 | 9.8 | 154 |
|  | 2-day | 4.6 | -2.8 | 12.5 | 142 |
|  |  |  |  |  |  |
| **Cr** | 0-day | 1.2 | -1.4 | 4.0 | 207 |
|  | 1-day | 2.6 | -1.0 | 6.2 | 188 |
|  | 2-day | 0.1 | -3.5 | 3.9 | 168 |
|  |  |  |  |  |  |
| **Cu** | 0-day | 1.8 | -0.3 | 4.0 | 184 |
|  | 1-day | 0.3 | -1.8 | 2.6 | 163 |
|  | 2-day | 0.7 | -1.2 | 2.7 | 146 |
|  |  |  |  |  |  |
| **Fe** | 0-day | 0.8 | -3.8 | 5.7 | 212 |
|  | 1-day | 7.5 | 1.5 | 13.9 | 191 |
|  | 2-day | 3.0 | -2.9 | 9.2 | 173 |
|  |  |  |  |  |  |
| **Mg** | 0-day | 2.3 | -3.3 | 8.2 | 179 |
|  | 1-day | 7.7 | 1.8 | 13.9 | 163 |
|  | 2-day | 5.6 | -0.5 | 12.0 | 146 |
|  |  |  |  |  |  |
| **Mn** | 0-day | -1.2 | -4.7 | 2.4 | 204 |
|  | 1-day | -0.5 | -4.7 | 3.9 | 182 |
|  | 2-day | 2.4 | -2.7 | 7.7 | 164 |
|  |  |  |  |  |  |
| **Ni** | 0-day | 1.1 | -2.7 | 5.1 | 173 |
|  | 1-day | 1.5 | -2.8 | 6.1 | 153 |
|  | 2-day | 2.4 | -1.9 | 6.8 | 138 |
|  |  |  |  |  |  |
| **S** | 0-day | 3.1 | -1.7 | 8.2 | 214 |
|  | 1-day | 2.5 | -2.9 | 8.2 | 192 |
|  | 2-day | 3.3 | -2.2 | 9.1 | 173 |

**Supplemental Information, Table S6.** *Continued.*

| **Exposure** | **Exposure** | **% Change** | **Lower** | **Upper** | **Total Number of** |
| --- | --- | --- | --- | --- | --- |
| **Metric** | **Lag Period** | **per IQR** | **95% CI** | **95% CI** | **Observations** |
| **Sb** | 0-day | 1.7 | -14.7 | 21.3 | 57 |
|  | 1-day | False Convergence | | | |
|  | 2-day | 2.7 | -12.8 | 20.9 | 50 |
|  |  |  |  |  |  |
| **Sr** | 0-day | 1.4 | -3.5 | 6.6 | 107 |
|  | 1-day | -1.5 | -6.2 | 3.4 | 97 |
|  | 2-day | 1.4 | -1.8 | 4.6 | 88 |
|  |  |  |  |  |  |
| **V** | 0-day | 5.0 | -2.9 | 13.5 | 130 |
|  | 1-day | 0.8 | -6.1 | 8.1 | 120 |
|  | **2-day** | **7.6** | **0.1** | **15.8** | **110** |
|  |  |  |  |  |  |
| **Zn** | 0-day | -0.9 | -4.3 | 2.5 | 208 |
|  | 1-day | 1.7 | -1.8 | 5.2 | 188 |
|  | 2-day | 1.9 | -1.9 | 5.8 | 169 |

**Supplemental Information, Table S7.** Effects of metal exposure concentration (pg/µg) on the percent change in FeNO as modified by medication use (none, any, corticosteroids, beta-agonists, stimulants) for 0-, 1- and 2-day lag periods. Models were adjusted by the fixed effects (temperature, sex, the presence of allergies, eczema before the age of 2, occurrence of an asthma attack in the first year of life, use of beta-agonists, use of corticosteroids and parental asthma) as well as random subject and day of study factors. *Note: Medication use was evaluated as possible effect modifiers by including an interaction term with personal trace metal exposure concentration. Models with and without this interaction term were compared with the likelihood-ratio statistic using full maximum likelihood. The restricted maximum likelihood approach was used to estimate model parameters.*

| **Al** | **Exposure**  **Lag Period** | **% Change per IQR** | **Lower 95% CI** | **Upper 95% CI** | **Total Observations** | **Interaction Term** |
| --- | --- | --- | --- | --- | --- | --- |
| **0 day lag** | **All Children** | 4.2 | -1.1 | 9.7 | 208 |  |
|  | **No Medications** | 5.5 | -2.4 | 14.1 | 89 | p = 0.80 |
|  | **Any Medication Use** | 2.8 | -4.3 | 10.4 | 119 |  |
|  | **No Corticosteroids** | 5.0 | -2.3 | 12.9 | 96 | p = 0.44 |
|  | **Irregular Corticosteroid Use** | 3.5 | -4.4 | 11.9 | 112 |  |
|  | **Regular Corticosteroid Use** | 7.2 | -1.3 | 16.3 | 48 |  |
|  | **No Beta-agonists** | False Convergence | | | | p = 0.19 |
|  | **Beta-agonist Use** | -1.5 | -16.7 | 16.5 | 33 |  |
|  | | | | | |  |
| **1 day lag** | **All Children** | **10.3** | **4.2** | **16.6** | **188** |  |
|  | **No Medications** | **14.0** | **4.3** | **24.7** | **80** | p = 0.77 |
|  | **Any Medication Use** | **7.7** | **0.3** | **15.6** | **108** |  |
|  | **No Corticosteroids** | **13.6** | **5.0** | **22.8** | **87** | p = 0.38 |
|  | **Irregular Corticosteroid Use** | 7.5 | -0.5 | 16.1 | 101 |  |
|  | **Regular Corticosteroid Use** | **12.8** | **4.9** | **21.3** | **43** |  |
|  | **No Beta-agonists** | False Convergence | | | | **p = 0.01** |
|  | **Beta-agonist Use** | 6.8 | -6.1 | 21.5 | 31 |  |
|  | | | | | |  |
| **2 day lag** | **All Children** | **6.0** | **0.0** | **12.5** | **169** |  |
|  | **No Medications** | 4.1 | -5.3 | 14.6 | 70 | p = 0.59 |
|  | **Any Medication Use** | 6.0 | -1.0 | 13.5 | 99 |  |
|  | **No Corticosteroids** | 3.2 | -4.8 | 11.8 | 77 | p = 0.74 |
|  | **Irregular Corticosteroid Use** | 7.2 | -0.3 | 15.2 | 92 |  |
|  | **Regular Corticosteroid Use** | **7.3** | **0.4** | **14.7** | **41** |  |
|  | **No Beta-agonists** | False Convergence | | | | p = 0.06 |
|  | **Beta-agonist Use** | 6.9 | -8.5 | 24.8 | 30 |  |

**Supplemental Information, Table S7.** *Continued.*

| **As** | **Exposure**  **Lag Period** | **% Change per IQR** | **Lower 95% CI** | **Upper 95% CI** | **Total Observations** | **Interaction Term** |
| --- | --- | --- | --- | --- | --- | --- |
| **0 day lag** | **All Children** | 3.5 | -6.6 | 14.7 | 84 |  |
|  | **No Medications** | 6.3 | -7.3 | 22.0 | 41 | p = 0.92 |
|  | **Any Medication Use** | False Convergence | | | |  |
|  | **No Corticosteroids** | 4.0 | -8.6 | 18.5 | 44 | p = 0.46 |
|  | **Irregular Corticosteroid Use** | 6.4 | -8.1 | 23.2 | 40 |  |
|  | **Regular Corticosteroid Use** | False Convergence | | | |  |
|  | **No Beta-agonists** | False Convergence | | | | p = 0.51 |
|  | **Beta-agonist Use** | 11.9 | -28.2 | 74.4 | 14 |  |
|  | | | | | |  |
| **1 day lag** | **All Children** | 8.4 | -2.4 | 20.3 | 73 |  |
|  | **No Medications** | **15.0** | **8.0** | **22.5** | **35** | p = 0.77 |
|  | **Any Medication Use** | 4.5 | -13.9 | 26.8 | 38 |  |
|  | **No Corticosteroids** | **16.9** | **9.1** | **25.4** | **38** | p = 0.80 |
|  | **Irregular Corticosteroid Use** | 8.2 | -12.7 | 34.0 | 35 |  |
|  | **Regular Corticosteroid Use** | **13.2** | **3.3** | **24.1** | **15** |  |
|  | **No Beta-agonists** | False Convergence | | | | False Convergence |
|  | **Beta-agonist Use** | False Convergence | | | |  |
|  | | | | | |  |
| **2 day lag** | **All Children** | 4.3 | -6.9 | 16.8 | 63 |  |
|  | **No Medications** | 3.9 | -8.9 | 18.6 | 26 | p = 0.48 |
|  | **Any Medication Use** | 0.1 | -18.3 | 22.6 | 37 |  |
|  | **No Corticosteroids** | 0.4 | -13.3 | 16.3 | 30 | p = 0.71 |
|  | **Irregular Corticosteroid Use** | 4.2 | -15.9 | 29.1 | 33 |  |
|  | **Regular Corticosteroid Use** | False Convergence | | | |  |
|  | **No Beta-agonists** | False Convergence | | | | False Convergence |
|  | **Beta-agonist Use** | False Convergence | | | |  |

**Supplemental Information, Table S7.** *Continued.*

| **Ba** | **Exposure**  **Lag Period** | **% Change per IQR** | **Lower 95% CI** | **Upper 95% CI** | **Total Observations** | **Interaction Term** |
| --- | --- | --- | --- | --- | --- | --- |
| **0 day lag** | **All Children** | **8.9** | **2.8** | **15.4** | **169** |  |
|  | **No Medications** | **10.0** | **1.1** | **19.7** | **74** | p = 0.74 |
|  | **Any Medication Use** | **8.2** | **0.3** | **16.8** | **95** |  |
|  | **No Corticosteroids** | **8.3** | **0.1** | **17.1** | **80** | p = 0.60 |
|  | **Irregular Corticosteroid Use** | **8.7** | **0.4** | **17.8** | **89** |  |
|  | **Regular Corticosteroid Use** | 4.7 | -10.7 | 22.8 | 39 |  |
|  | **No Beta-agonists** | False Convergence | | | | p = 0.39 |
|  | **Beta-agonist Use** | 10.6 | -8.6 | 33.7 | 25 |  |
|  | | | | | |  |
| **1 day lag** | **All Children** | 3.2 | -3.1 | 9.8 | 154 |  |
|  | **No Medications** | 5.8 | -4.4 | 17.1 | 65 | p = 0.79 |
|  | **Any Medication Use** | 2.1 | -6.5 | 11.6 | 89 |  |
|  | **No Corticosteroids** | 4.2 | -3.1 | 12.0 | 71 | p = 0.92 |
|  | **Irregular Corticosteroid Use** | 3.0 | -7.0 | 14.1 | 83 |  |
|  | **Regular Corticosteroid Use** | 8.4 | -6.2 | 25.2 | 37 |  |
|  | **No Beta-agonists** | False Convergence | | | | p = 0.61 |
|  | **Beta-agonist Use** | 7.5 | -6.0 | 22.9 | 24 |  |
|  | | | | | |  |
| **2 day lag** | **All Children** | 4.6 | -2.8 | 12.5 | 142 |  |
|  | **No Medications** | 3.0 | -6.4 | 13.3 | 60 | p = 0.83 |
|  | **Any Medication Use** | 4.1 | -4.7 | 13.7 | 82 |  |
|  | **No Corticosteroids** | 2.9 | -6.0 | 12.6 | 66 | p = 0.83 |
|  | **Irregular Corticosteroid Use** | 6.8 | -4.3 | 19.1 | 76 |  |
|  | **Regular Corticosteroid Use** | 15.0 | -1.1 | 33.7 | 35 |  |
|  | **No Beta-agonists** | False Convergence | | | | p = 0.81 |
|  | **Beta-agonist Use** | -1.1 | -18.6 | 20.1 | 24 |  |

**Supplemental Information, Table S7.** *Continued.*

| **Cr** | **Exposure**  **Lag Period** | **% Change per IQR** | **Lower 95% CI** | **Upper 95% CI** | **Total Observations** | **Interaction Term** |
| --- | --- | --- | --- | --- | --- | --- |
| **0 day lag** | **All Children** | 1.8 | -0.3 | 4.0 | 184 |  |
|  | **No Medications** | 2.6 | -1.9 | 7.2 | 80 | p = 0.86 |
|  | **Any Medication Use** | 1.8 | -0.9 | 4.6 | 104 |  |
|  | **No Corticosteroids** | 2.1 | -2.3 | 6.7 | 85 | p = 0.74 |
|  | **Irregular Corticosteroid Use** | 2.2 | -1.0 | 5.6 | 99 |  |
|  | **Regular Corticosteroid Use** | False Convergence | | | |  |
|  | **No Beta-agonists** | False Convergence | | | | p = 0.35 |
|  | **Beta-agonist Use** | 8.3 | -5.0 | 23.5 | 30 |  |
|  | | | | | |  |
| **1 day lag** | **All Children** | 0.3 | -1.8 | 2.6 | 163 |  |
|  | **No Medications** | 1.2 | -3.0 | 5.4 | 73 | p = 0.75 |
|  | **Any Medication Use** | 0.1 | -2.8 | 3.2 | 90 |  |
|  | **No Corticosteroids** | 1.1 | -3.0 | 5.3 | 78 | p = 0.85 |
|  | **Irregular Corticosteroid Use** | 0.3 | -3.2 | 3.9 | 85 |  |
|  | **Regular Corticosteroid Use** | 1.5 | -1.9 | 4.9 | 36 |  |
|  | **No Beta-agonists** | False Convergence | | | | p = 0.98 |
|  | **Beta-agonist Use** | 7.5 | -3.0 | 19.2 | 27 |  |
|  | | | | | |  |
| **2 day lag** | **All Children** | 0.7 | -1.2 | 2.7 | 146 |  |
|  | **No Medications** | 1.7 | -1.0 | 4.4 | 65 | p = 0.40 |
|  | **Any Medication Use** | 0.4 | -2.4 | 3.3 | 81 |  |
|  | **No Corticosteroids** | 1.9 | -0.9 | 4.9 | 69 | p = 0.50 |
|  | **Irregular Corticosteroid Use** | 0.6 | -2.7 | 3.9 | 77 |  |
|  | **Regular Corticosteroid Use** | 0.2 | -3.6 | 4.1 | 34 |  |
|  | **No Beta-agonists** | False Convergence | | | | p = 0.67 |
|  | **Beta-agonist Use** | -0.5 | -14.9 | 16.5 | 25 |  |

**Supplemental Information, Table S7.** *Continued.*

| **Cu** | **Exposure**  **Lag Period** | **% Change per IQR** | **Lower 95% CI** | **Upper 95% CI** | **Total Observations** | **Interaction Term** |
| --- | --- | --- | --- | --- | --- | --- |
| **0 day lag** | **All Children** | 1.2 | -1.4 | 4.0 | 207 |  |
|  | **No Medications** | False Convergence | | | | p = 0.80 |
|  | **Any Medication Use** | 1.5 | -3.4 | 6.6 | 117 |  |
|  | **No Corticosteroids** | False Convergence | | | | p = 0.93 |
|  | **Irregular Corticosteroid Use** | 2.3 | -2.8 | 7.7 | 110 |  |
|  | **Regular Corticosteroid Use** | False Convergence | | | |  |
|  | **No Beta-agonists** | False Convergence | | | | p = 0.51 |
|  | **Beta-agonist Use** | -1.1 | -5.4 | 3.5 | 33 |  |
|  | | | | | |  |
| **1 day lag** | **All Children** | 2.6 | -1.0 | 6.2 | 188 |  |
|  | **No Medications** | 2.7 | -0.8 | 6.4 | 80 | p = 0.66 |
|  | **Any Medication Use** | 1.5 | -4.5 | 8.0 | 108 |  |
|  | **No Corticosteroids** | 2.4 | -0.9 | 5.9 | 87 | p = 0.51 |
|  | **Irregular Corticosteroid Use** | 2.7 | -3.7 | 9.7 | 101 |  |
|  | **Regular Corticosteroid Use** | 8.0 | -2.4 | 19.6 | 43 |  |
|  | **No Beta-agonists** | False Convergence | | | | p = 0.90 |
|  | **Beta-agonist Use** | -3.3 | -13.3 | 7.9 | 30 |  |
|  | | | | | |  |
| **2 day lag** | **All Children** | 0.1 | -3.5 | 3.9 | 168 |  |
|  | **No Medications** | 3.5 | -0.8 | 7.9 | 70 | p = 0.09 |
|  | **Any Medication Use** | -2.5 | -8.5 | 3.9 | 98 |  |
|  | **No Corticosteroids** | 2.2 | -1.7 | 6.3 | 77 | p = 0.11 |
|  | **Irregular Corticosteroid Use** | -1.1 | -7.5 | 5.7 | 91 |  |
|  | **Regular Corticosteroid Use** | 6.0 | -3.4 | 16.4 | 40 |  |
|  | **No Beta-agonists** | False Convergence | | | | p = 0.88 |
|  | **Beta-agonist Use** | -0.9 | -13.4 | 13.4 | 29 |  |

**Supplemental Information, Table S7.** *Continued.*

| **Fe** | **Exposure**  **Lag Period** | **% Change per IQR** | **Lower 95% CI** | **Upper 95% CI** | **Total Observations** | **Interaction Term** |
| --- | --- | --- | --- | --- | --- | --- |
| **0 day lag** | **All Children** | 0.8 | -3.8 | 5.7 | 212 |  |
|  | **No Medications** | 0.3 | -6.6 | 7.8 | 92 | p = 0.84 |
|  | **Any Medication Use** | 0.9 | -4.8 | 7.0 | 120 |  |
|  | **No Corticosteroids** | -1.8 | -7.2 | 4.0 | 99 | p = 0.45 |
|  | **Irregular Corticosteroid Use** | 3.9 | -2.9 | 11.3 | 113 |  |
|  | **Regular Corticosteroid Use** | False Convergence | | | |  |
|  | **No Beta-agonists** | False Convergence | | | | p = 0.09 |
|  | **Beta-agonist Use** | -6.3 | -12.2 | 0.0 | 34 |  |
|  | | | | | |  |
| **1 day lag** | **All Children** | **7.5** | **1.5** | **13.9** | **191** |  |
|  | **No Medications** | **9.3** | **0.2** | **19.2** | **82** | p = 0.82 |
|  | **Any Medication Use** | 5.7 | -1.6 | 13.4 | 109 |  |
|  | **No Corticosteroids** | **8.4** | **0.5** | **17.0** | **89** | p = 0.85 |
|  | **Irregular Corticosteroid Use** | 6.5 | -2.0 | 15.8 | 102 |  |
|  | **Regular Corticosteroid Use** | **11.3** | **1.3** | **22.4** | **44** |  |
|  | **No Beta-agonists** | False Convergence | | | | **p = 0.04** |
|  | **Beta-agonist Use** | -2.7 | -14.6 | 11.0 | 31 |  |
|  | | | | | |  |
| **2 day lag** | **All Children** | 3.0 | -2.9 | 9.2 | 173 |  |
|  | **No Medications** | 6.4 | -1.9 | 15.4 | 73 | p = 0.38 |
|  | **Any Medication Use** | 0.4 | -6.9 | 8.2 | 100 |  |
|  | **No Corticosteroids** | 5.2 | -1.9 | 12.8 | 80 | p = 0.73 |
|  | **Irregular Corticosteroid Use** | 1.5 | -6.7 | 10.3 | 93 |  |
|  | **Regular Corticosteroid Use** | 3.6 | -7.6 | 16.1 | 42 |  |
|  | **No Beta-agonists** | False Convergence | | | | p = 0.14 |
|  | **Beta-agonist Use** | -0.8 | -13.5 | 13.7 | 30 |  |

**Supplemental Information, Table S7.** *Continued.*

| **Mg** | **Exposure**  **Lag Period** | **% Change per IQR** | **Lower 95% CI** | **Upper 95% CI** | **Total Observations** | **Interaction Term** |
| --- | --- | --- | --- | --- | --- | --- |
| **0 day lag** | **All Children** | 2.3 | -3.3 | 8.2 | 179 |  |
|  | **No Medications** | 2.7 | -3.8 | 9.6 | 82 | p = 0.75 |
|  | **Any Medication Use** | 2.7 | -5.4 | 11.6 | 97 |  |
|  | **No Corticosteroids** | False Convergence | | | | p = 0.96 |
|  | **Irregular Corticosteroid Use** | 2.8 | -6.0 | 12.4 | 91 |  |
|  | **Regular Corticosteroid Use** | 1.1 | -10.4 | 14.1 | 44 |  |
|  | **No Beta-agonists** | False Convergence | | | | p = 0.68 |
|  | **Beta-agonist Use** | -2.8 | -18.8 | 16.5 | 30 |  |
|  | | | | | |  |
| **1 day lag** | **All Children** | **7.7** | **1.8** | **13.9** | **163** |  |
|  | **No Medications** | **11.6** | **3.7** | **20.1** | **74** | p = 0.69 |
|  | **Any Medication Use** | 4.7 | -3.2 | 13.2 | 89 |  |
|  | **No Corticosteroids** | **10.9** | **3.5** | **18.8** | **80** | p = 0.87 |
|  | **Irregular Corticosteroid Use** | 4.4 | -3.9 | 13.4 | 83 |  |
|  | **Regular Corticosteroid Use** | 8.3 | -1.7 | 19.3 | 41 |  |
|  | **No Beta-agonists** | False Convergence | | | | p = 0.81 |
|  | **Beta-agonist Use** | 1.9 | -10.9 | 16.6 | 28 |  |
|  | | | | | |  |
| **2 day lag** | **All Children** | 5.6 | -0.5 | 12.0 | 146 |  |
|  | **No Medications** | **6.6** | **0.6** | **13.0** | **63** | p = 0.96 |
|  | **Any Medication Use** | 4.3 | -4.6 | 14.1 | 83 |  |
|  | **No Corticosteroids** | **6.3** | **0.2** | **12.8** | **69** | p = 0.98 |
|  | **Irregular Corticosteroid Use** | 5.1 | -4.1 | 15.2 | 77 |  |
|  | **Regular Corticosteroid Use** | 4.8 | -4.4 | 15.0 | 39 |  |
|  | **No Beta-agonists** | False Convergence | | | | p = 0.90 |
|  | **Beta-agonist Use** | -1.6 | -18.8 | 19.2 | 27 |  |

**Supplemental Information, Table S7.** *Continued.*

| **Mn** | **Exposure**  **Lag Period** | **% Change per IQR** | **Lower 95% CI** | **Upper 95% CI** | **Total Observations** | **Interaction Term** |
| --- | --- | --- | --- | --- | --- | --- |
| **0 day lag** | **All Children** | -1.2 | -4.7 | 2.4 | 204 |  |
|  | **No Medications** | -1.4 | -5.2 | 2.6 | 88 | p = 0.72 |
|  | **Any Medication Use** | -0.8 | -7.2 | 6.2 | 116 |  |
|  | **No Corticosteroids** | -1.8 | -5.0 | 1.5 | 95 | p = 0.64 |
|  | **Irregular Corticosteroid Use** | 1.1 | -6.7 | 9.6 | 109 |  |
|  | **Regular Corticosteroid Use** | False Convergence | | | |  |
|  | **No Beta-agonists** | False Convergence | | | | p = 0.08 |
|  | **Beta-agonist Use** | -4.8 | -10.5 | 1.3 | 31 |  |
|  | | | | | |  |
| **1 day lag** | **All Children** | -0.5 | -4.7 | 3.9 | 182 |  |
|  | **No Medications** | -0.4 | -4.6 | 3.9 | 77 | p = 0.68 |
|  | **Any Medication Use** | 0.5 | -8.3 | 10.2 | 105 |  |
|  | **No Corticosteroids** | -0.4 | -4.1 | 3.5 | 84 | p = 0.65 |
|  | **Irregular Corticosteroid Use** | -0.3 | -9.3 | 9.6 | 98 |  |
|  | **Regular Corticosteroid Use** | 7.7 | -5.5 | 22.7 | 43 |  |
|  | **No Beta-agonists** | False Convergence | | | | p = 0.29 |
|  | **Beta-agonist Use** | 3.7 | -8.2 | 17.2 | 29 |  |
|  | | | | | |  |
| **2 day lag** | **All Children** | 2.4 | -2.7 | 7.7 | 164 |  |
|  | **No Medications** | 2.9 | -2.4 | 8.5 | 68 | p = 0.88 |
|  | **Any Medication Use** | 2.7 | -5.5 | 11.6 | 96 |  |
|  | **No Corticosteroid** | 2.5 | -2.5 | 7.9 | 75 | p = 0.91 |
|  | **Irregular Corticosteroid Use** | 3.2 | -6.0 | 13.4 | 89 |  |
|  | **Regular Corticosteroid Use** | 8.6 | -2.1 | 20.5 | 41 |  |
|  | **No Beta-agonists** | False Convergence | | | | **p = 0.05** |
|  | **Beta-agonist Use** | 5.6 | -13.0 | 28.2 | 27 |  |

**Supplemental Information, Table S7.** *Continued.*

| **Ni** | **Exposure**  **Lag Period** | **% Change per IQR** | **Lower 95% CI** | **Upper 95% CI** | **Total Observations** | **Interaction Term** |
| --- | --- | --- | --- | --- | --- | --- |
| **0 day lag** | **All Children** | 1.1 | -2.7 | 5.1 | 173 |  |
|  | **No Medications** | False Convergence | | | | p = 0.81 |
|  | **Any Medication Use** | 2.2 | -3.8 | 8.6 | 99 |  |
|  | **No Corticosteroids** |  | | | | p = 0.97 |
|  | **Irregular Corticosteroid Use** | 2.6 | -3.7 | 9.4 | 94 |  |
|  | **Regular Corticosteroid Use** | 2.4 | -8.4 | 14.5 | 39 |  |
|  | **No Beta-agonists** | False Convergence | | | | p = 0.25 |
|  | **Beta-agonist Use** | 3.2 | -6.0 | 13.3 | 25 |  |
|  | | | | | |  |
| **1 day lag** | **All Children** | 1.5 | -2.8 | 6.1 | 153 |  |
|  | **No Medications** | 1.0 | -3.4 | 5.5 | 65 | p = 0.82 |
|  | **Any Medication Use** | 2.7 | -5.6 | 11.7 | 88 |  |
|  | **No Corticosteroids** | 1.1 | -2.9 | 5.2 | 70 | p = 0.93 |
|  | **Irregular Corticosteroid Use** | 2.3 | -7.1 | 12.6 | 83 |  |
|  | **Regular Corticosteroid Use** | False Convergence | | | |  |
|  | **No Beta-agonists** | False Convergence | | | | p = 0.36 |
|  | **Beta-agonist Use** | **12.6** | **2.4** | **23.9** | **23** |  |
|  | | | | | |  |
| **2 day lag** | **All Children** | 2.4 | -1.9 | 6.8 | 138 |  |
|  | **No Medications** | **4.1** | **0.5** | **7.8** | **59** | p = 0.17 |
|  | **Any Medication Use** | -1.9 | -10.7 | 7.8 | 79 |  |
|  | **No Corticosteroids** | **4.0** | **0.7** | **7.6** | **64** | p = 0.33 |
|  | **Irregular Corticosteroid Use** | -1.8 | -11.3 | 8.8 | 74 |  |
|  | **Regular Corticosteroid Use** | 2.1 | -13.9 | 21.1 | 32 |  |
|  | **No Beta-agonists** | False Convergence | | | | **p = 0.001** |
|  | **Beta-agonist Use** | 9.6 | -0.7 | 20.9 | 21 |  |

**Supplemental Information, Table S7.** *Continued.*

| **S** | **Exposure**  **Lag Period** | **% Change per IQR** | **Lower 95% CI** | **Upper 95% CI** | **Total Observations** | **Interaction Term** |
| --- | --- | --- | --- | --- | --- | --- |
| **0 day lag** | **All Children** | 3.1 | -1.7 | 8.2 | 214 |  |
|  | **No Medications** | 1.7 | -5.1 | 9.0 | 93 | p = 0.71 |
|  | **Any Medication Use** | 2.9 | -4.4 | 10.8 | 121 |  |
|  | **No Corticosteroids** | 2.1 | -4.4 | 9.1 | 100 | p = 0.80 |
|  | **Irregular Corticosteroid Use** | False Convergence | | | |  |
|  | **Regular Corticosteroid Use** | False Convergence | | | |  |
|  | **No Beta-agonists** | 0.6 | -15.8 | 20.1 | 34 | p = 0.73 |
|  | **Beta-agonist Use** | 2.3 | -2.9 | 7.8 | 194 |  |
|  | | | | | |  |
| **1 day lag** | **All Children** | 2.5 | -2.9 | 8.2 | 192 |  |
|  | **No Medications** | -0.4 | -7.0 | 6.6 | 83 | p = 0.46 |
|  | **Any Medication Use** | 3.0 | -5.1 | 11.8 | 109 |  |
|  | **No Corticosteroids** | 1.5 | -4.9 | 8.3 | 90 | p = 0.99 |
|  | **Irregular Corticosteroid Use** | 2.1 | -6.7 | 11.8 | 102 |  |
|  | **Regular Corticosteroid Use** | 7.4 | -4.9 | 21.3 | 44 |  |
|  | **No Beta-agonists** | False Convergence | | | | p = 0.08 |
|  | **Beta-agonist Use** | 4.2 | -8.5 | 18.6 | 31 |  |
|  | | | | | |  |
| **2 day lag** | **All Children** | 3.3 | -2.2 | 9.1 | 173 |  |
|  | **No Medications** | 3.6 | -3.7 | 11.4 | 73 | p = 0.76 |
|  | **Any Medication Use** | 2.2 | -5.3 | 10.4 | 100 |  |
|  | **No Corticosteroids** | 1.8 | -4.6 | 8.7 | 80 | p = 0.69 |
|  | **Irregular Corticosteroid Use** | 3.8 | -4.5 | 12.8 | 93 |  |
|  | **Regular Corticosteroid Use** | 6.6 | -2.6 | 16.5 | 42 |  |
|  | **No Beta-agonists** | False Convergence | | | | p = 0.11 |
|  | **Beta-agonist Use** | -4.2 | -18.8 | 13.1 | 30 |  |

**Supplemental Information, Table S7.** *Continued.*

| **Sb** | **Exposure**  **Lag Period** | **% Change per IQR** | **Lower 95% CI** | **Upper 95% CI** | **Total Observations** | **Interaction Term** |
| --- | --- | --- | --- | --- | --- | --- |
| **0 day lag** | **All Children** | 1.7 | -14.7 | 21.3 | 57 |  |
|  | **No Medications** | **39.7** | **4.0** | **87.7** | **24** | False Convergence |
|  | **Any Medication Use** | False Convergence | | | |  |
|  | **No Corticosteroids** | -1.7 | -16.1 | 15.2 | 17 | False Convergence |
|  | **Irregular Corticosteroid Use** | False Convergence | | | |  |
|  | **Regular Corticosteroid Use** | False Convergence | | | |  |
|  | **No Beta-agonists** | False Convergence | | | | p = 0.45 |
|  | **Beta-agonist Use** | 6.1 | -11.5 | 27.3 | 52 |  |
|  | | | | | |  |
| **1 day lag** | **All Children** | False Convergence | | | |  |
|  | **No Medications** | False Convergence | | | | False Convergence |
|  | **Any Medication Use** | False Convergence | | | |  |
|  | **No Corticosteroids** | False Convergence | | | | False Convergence |
|  | **Irregular Corticosteroid Use** | False Convergence | | | |  |
|  | **Regular Corticosteroid Use** | False Convergence | | | |  |
|  | **No Beta-agonists** | False Convergence | | | | False Convergence |
|  | **Beta-agonist Use** | False Convergence | | | |  |
|  | | | | | |  |
| **2 day lag** | **All Children** | 2.7 | -12.8 | 20.9 | 50 |  |
|  | **No Medications** | 4.5 | -18.4 | 33.8 | 24 | p = 0.54 |
|  | **Any Medication Use** | False Convergence | | | |  |
|  | **No Corticosteroids** | 4.0 | -16.9 | 30.3 | 25 | p = 0.61 |
|  | **Irregular Corticosteroid Use** | 1.7 | -18.6 | 27.1 | 25 |  |
|  | **Regular Corticosteroid Use** | False Convergence | | | |  |
|  | **No Beta-agonists** | False Convergence | | | | p = 0.79 |
|  | **Beta-agonist Use** | 3.3 | -32.4 | 57.9 | 8 |  |

**Supplemental Information, Table S7.** *Continued.*

| **Sr** | **Exposure**  **Lag Period** | **% Change per IQR** | **Lower 95% CI** | **Upper 95% CI** | **Total Observations** | **Interaction Term** |
| --- | --- | --- | --- | --- | --- | --- |
| **0 day lag** | **All Children** | 1.4 | -3.5 | 6.6 | 107 |  |
|  | **No Medications** | 5.1 | -6.1 | 17.6 | 41 | p = 0.93 |
|  | **Any Medication Use** | 1.9 | -5.1 | 9.3 | 66 |  |
|  | **No Corticosteroids** | 4.2 | -5.3 | 14.7 | 44 | False Convergence |
|  | **Irregular Corticosteroid Use** | False Convergence | | | |  |
|  | **Regular Corticosteroid Use** | False Convergence | | | |  |
|  | **No Beta-agonists** | False Convergence | | | | False Convergence |
|  | **Beta-agonist Use** | -47.7 | -57.5 | -35.5 | 17 |  |
|  | | | | | |  |
| **1 day lag** | **All Children** | -1.5 | -6.2 | 3.4 | 97 |  |
|  | **No Medications** | 0.6 | -8.7 | 10.7 | 36 | p = 0.61 |
|  | **Any Medication Use** | -2.5 | -8.6 | 4.1 | 61 |  |
|  | **No Corticosteroids** | 0.2 | -8.6 | 9.8 | 38 | p = 0.77 |
|  | **Irregular Corticosteroid Use** | -2.3 | -8.7 | 4.6 | 59 |  |
|  | **Regular Corticosteroid Use** | **8.6** | **1.1** | **16.6** | **21** |  |
|  | **No Beta-agonists** | False Convergence | | | | p = 0.23 |
|  | **Beta-agonist Use** | False Convergence | | | |  |
|  | | | | | |  |
| **2 day lag** | **All Children** | 1.4 | -1.8 | 4.6 | 88 |  |
|  | **No Medications** | 5.6 | -1.1 | 12.7 | 34 | False Convergence |
|  | **Any Medication Use** |  | | | |  |
|  | **No Corticosteroids** | 4.7 | -1.0 | 10.6 | 36 | False Convergence |
|  | **Irregular Corticosteroid Use** | -1.2 | -8.9 | 7.1 | 52 |  |
|  | **Regular Corticosteroid Use** | 3.0 | -14.8 | 24.4 | 19 |  |
|  | **No Beta-agonists** | False Convergence | | | | False Convergence |
|  | **Beta-agonist Use** | -0.9 | -6.8 | 5.3 | 15 |  |

**Supplemental Information, Table S7.** *Continued.*

| **V** | **Exposure**  **Lag Period** | **% Change per IQR** | **Lower 95% CI** | **Upper 95% CI** | **Total Observations** | **Interaction Term** |
| --- | --- | --- | --- | --- | --- | --- |
| **0 day lag** | **All Children** | 5.0 | -2.9 | 13.5 | 130 |  |
|  | **No Medications** | False Convergence | | | | p = 0.62 |
|  | **Any Medication Use** | 6.1 | -8.3 | 22.7 | 73 |  |
|  | **No Corticosteroids** |  | | | | p = 0.75 |
|  | **Irregular Corticosteroid Use** | 5.6 | -9.1 | 22.7 | 68 |  |
|  | **Regular Corticosteroid Use** | 9.6 | -14.7 | 40.7 | 26 |  |
|  | **No Beta-agonists** | False Convergence | | | | p = 0.64 |
|  | **Beta-agonist Use** | 22.7 | -32.6 | 123.4 | 19 |  |
|  | | | | | |  |
| **1 day lag** | **All Children** | 0.8 | -6.1 | 8.1 | 120 |  |
|  | **No Medications** | 3.2 | -4.1 | 11.2 | 49 | p = 0.64 |
|  | **Any Medication Use** | -0.2 | -11.1 | 12.1 | 71 |  |
|  | **No Corticosteroids** | 3.2 | -3.3 | 10.1 | 54 | p = 0.77 |
|  | **Irregular Corticosteroid Use** | -1.5 | -13.5 | 12.2 | 66 |  |
|  | **Regular Corticosteroid Use** | -4.1 | -15.8 | 9.2 | 26 |  |
|  | **No Beta-agonists** | False Convergence | | | | p = 0.19 |
|  | **Beta-agonist Use** | 30.3 | -8.7 | 86.0 | 18 |  |
|  | | | | | |  |
| **2 day lag** | **All Children** | **7.6** | **0.1** | **15.8** | **110** |  |
|  | **No Medications** | **7.1** | **0.4** | **14.3** | **47** | p = 0.87 |
|  | **Any Medication Use** | 10.7 | -2.6 | 25.9 | 63 |  |
|  | **No Corticosteroids** | 5.8 | -0.2 | 12.1 | 51 | p = 0.78 |
|  | **Irregular Corticosteroid Use** | 9.9 | -3.7 | 25.4 | 59 |  |
|  | **Regular Corticosteroid Use** | 6.9 | -5.7 | 21.2 | 25 |  |
|  | **No Beta-agonists** | False Convergence | | | | **p = 0.01** |
|  | **Beta-agonist Use** | 2.5 | -50.0 | 110.2 | 17 |  |

**Supplemental Information, Table S7.** *Continued.*

| **Zn** | **Exposure**  **Lag Period** | **% Change per IQR** | **Lower 95% CI** | **Upper 95% CI** | **Total Observations** | **Interaction Term** |
| --- | --- | --- | --- | --- | --- | --- |
| **0 day lag** | **All Children** | -0.9 | -4.3 | 2.5 | 208 |  |
|  | **No Medications** | False Convergence | | | | p = 0.77 |
|  | **Any Medication Use** | -1.5 | -6.4 | 3.7 | 119 |  |
|  | **No Corticosteroids** | False Convergence | | | | p = 0.92 |
|  | **Irregular Corticosteroid Use** | -0.9 | -5.8 | 4.3 | 112 |  |
|  | **Regular Corticosteroid Use** | False Convergence | | | |  |
|  | **No Beta-agonists** | False Convergence | | | | p = 0.76 |
|  | **Beta-agonist Use** | -2.6 | -10.3 | 5.7 | 33 |  |
|  | | | | | |  |
| **1 day lag** | **All Children** | 1.7 | -1.8 | 5.2 | 188 |  |
|  | **No Medications** | 1.2 | -3.1 | 5.6 | 80 | p = 0.93 |
|  | **Any Medication Use** | 1.3 | -3.6 | 6.5 | 108 |  |
|  | **No Corticosteroids** | 1.2 | -2.5 | 5.1 | 87 | p = 0.94 |
|  | **Irregular Corticosteroid Use** | 1.5 | -3.5 | 6.7 | 101 |  |
|  | **Regular Corticosteroid Use** | 2.8 | -6.3 | 12.7 | 43 |  |
|  | **No Beta-agonists** | False Convergence | | | | p = 0.50 |
|  | **Beta-agonist Use** | 3.2 | -4.2 | 11.0 | 30 |  |
|  | | | | | |  |
| **2 day lag** | **All Children** | 1.9 | -1.9 | 5.8 | 169 |  |
|  | **No Medications** | 1.0 | -2.5 | 4.7 | 70 | p = 0.94 |
|  | **Any Medication Use** | 1.9 | -4.0 | 8.1 | 99 |  |
|  | **No Corticosteroids** | 0.8 | -2.5 | 4.2 | 77 | p = 0.98 |
|  | **Irregular Corticosteroid Use** | 2.5 | -3.4 | 8.7 | 92 |  |
|  | **Regular Corticosteroid Use** | **10.4** | **1.0** | **20.6** | **41** |  |
|  | **No Beta-agonists** | False Convergence | | | | p = 0.89 |
|  | **Beta-agonist Use** | -1.3 | -16.0 | 15.9 | 29 |  |

**Supplemental Information, Table S8.** Effects of metal exposure concentration (pg/µg) on the percent change in FeNO as modified by dust, mould, pollen and fur allergies for 0-, 1- and 2-day lag periods. The mixed models included a random effect on subject, time variables (indicators for day 1-10 of the study), a first order autoregressive error, and were adjusted for the fixed effects of temperature, sex, the presence of allergies, eczema before the age of 2, occurrence of an asthma attack in previous 12 months, use of beta-agonists, use of corticosteroids and parental asthma. *Note: Presence of an allergy were evaluated as possible effect modifiers by including an interaction term with personal trace metal exposure concentration. Models with and without this interaction term were compared with the likelihood-ratio statistic using full maximum likelihood. The restricted maximum likelihood approach was used to estimate model parameters.*

| **Al** | **Exposure**  **Lag Period** | **% Change per IQR** | **Lower 95% CI** | **Upper 95% CI** | **Total Observations** | **Interaction Term** |
| --- | --- | --- | --- | --- | --- | --- |
| **0 day lag** | **All Children** | 4.2 | -1.1 | 9.7 | 208 |  |
|  | **No Allergy** | 3.9 | -6.2 | 15.1 | 58 | p = 0.89 |
|  | **Presence of Any Allergy** | False Convergence | | | |  |
|  | **No Dust Allergy** | 5.5 | -2.7 | 14.5 | 93 | p = 0.77 |
|  | **Dust Allergy** | 2.9 | -3.9 | 10.1 | 115 |  |
|  | **No Mould Allergy** | 4.2 | -1.8 | 10.5 | 166 | p = 0.58 |
|  | **Mould Allergy** | 3.0 | -11.1 | 19.4 | 42 |  |
|  | **No Pollen Allergy** | **7.2** | **0.6** | **14.2** | **129** | p = 0.39 |
|  | **Pollen Allergy** | -1.1 | -11.6 | 10.6 | 79 |  |
|  | **No Fur Allergy** | 5.4 | -0.9 | 12.0 | 123 | p = 0.80 |
|  | **Fur Allergy** | 2.0 | -9.4 | 14.9 | 85 |  |
|  | | | | | |  |
| **1 day lag** | **All Children** | **10.3** | **4.2** | **16.6** | **188** |  |
|  | **No Allergy** | 10.0 | -1.0 | 22.1 | 56 | p = 0.66 |
|  | **Presence of Any Allergy** | **8.8** | **1.5** | **16.6** | **132** |  |
|  | **No Dust Allergy** | **13.1** | **3.9** | **23.2** | **87** | p = 0.09 |
|  | **Dust Allergy** | 3.6 | -3.8 | 11.5 | 101 |  |
|  | **No Mould Allergy** | **9.2** | **2.8** | **16.1** | **155** | p = 0.34 |
|  | **Mould Allergy** | **21.6** | **2.2** | **44.6** | **33** |  |
|  | **No Pollen Allergy** | **10.3** | **3.6** | **17.4** | **115** | p = 0.54 |
|  | **Pollen Allergy** | 12.4 | -0.5 | 26.9 | 73 |  |
|  | **No Fur Allergy** | **7.7** | **0.8** | **15.1** | **114** | p = 0.13 |
|  | **Fur Allergy** | **20.4** | **6.2** | **36.5** | **74** |  |
|  | | | | | |  |
| **2 day lag** | **All Children** | **6.0** | **0.0** | **12.5** | **169** |  |
|  | **No Allergy** | 6.2 | 0.0 | 12.7 | 51 | p = 0.13 |
|  | **Presence of Any Allergy** | 1.0 | -6.0 | 8.5 | 118 |  |
|  | **No Dust Allergy** | 7.3 | -1.9 | 17.3 | 78 | p = 0.45 |
|  | **Dust Allergy** | 1.4 | -5.6 | 9.0 | 91 |  |
|  | **No Mould Allergy** | 4.2 | -1.9 | 10.7 | 138 | **p = 0.03** |
|  | **Mould Allergy** | **27.1** | **10.2** | **46.6** | **31** |  |
|  | **No Pollen Allergy** | **6.3** | **0.5** | **12.4** | **100** | p = 0.13 |
|  | **Pollen Allergy** | 5.1 | -8.8 | 21.2 | 69 |  |
|  | **No Fur Allergy** | 5.5 | -0.4 | 11.8 | 101 | p = 0.77 |
|  | **Fur Allergy** | 10.8 | -4.5 | 28.5 | 68 |  |

**Supplemental Information, Table S8.** *Continued.*

| **As** | **Exposure**  **Lag Period** | **% Change per IQR** | **Lower 95% CI** | **Upper 95% CI** | **Total Observations** | **Interaction Term** |
| --- | --- | --- | --- | --- | --- | --- |
| **0 day lag** | **All Children** | 3.5 | -6.6 | 14.7 | 84 |  |
|  | **No Allergy** | -3.0 | -32.0 | 38.5 | 29 | p = 0.60 |
|  | **Presence of Any Allergy** | False Convergence | | | |  |
|  | **No Dust Allergy** | 4.3 | -19.9 | 35.8 | 43 | p = 0.29 |
|  | **Dust Allergy** | -0.4 | -10.6 | 11.1 | 41 |  |
|  | **No Mould Allergy** | 4.2 | -7.2 | 17.0 | 70 | p = 0.96 |
|  | **Mould Allergy** | -22.5 | -35.2 | -7.4 | 14 |  |
|  | **No Pollen Allergy** | 1.2 | -11.7 | 16.1 | 50 | p = 0.24 |
|  | **Pollen Allergy** | False Convergence | | | |  |
|  | **No Fur Allergy** | 4.8 | -8.9 | 20.6 | 54 | p = 0.71 |
|  | **Fur Allergy** | False Convergence | | | |  |
|  | | | | | |  |
| **1 day lag** | **All Children** | 8.4 | -2.4 | 20.3 | 73 |  |
|  | **No Allergy** | False Convergence | | | | p = 0.11 |
|  | **Presence of Any Allergy** | 7.6 | -3.0 | 19.4 | 46 |  |
|  | **No Dust Allergy** | False Convergence | | | | p = 0.10 |
|  | **Dust Allergy** | False Convergence | | | |  |
|  | **No Mould Allergy** | 6.2 | -5.2 | 19.1 | 62 | p = 0.39 |
|  | **Mould Allergy** | False Convergence | | | |  |
|  | **No Pollen Allergy** | False Convergence | | | | p = 0.62 |
|  | **Pollen Allergy** | 8.8 | -12.1 | 34.6 | 30 |  |
|  | **No Fur Allergy** | False Convergence | | | | p = 0.48 |
|  | **Fur Allergy** | 8.2 | -13.6 | 35.4 | 23 |  |
|  | | | | | |  |
| **2 day lag** | **All Children** | 4.3 | -6.9 | 16.8 | 63 |  |
|  | **No Allergy** | False Convergence | | | | p = 0.46 |
|  | **Presence of Any Allergy** | 3.7 | -8.9 | 18.1 | 39 |  |
|  | **No Dust Allergy** | 7.0 | -6.8 | 22.8 | 33 | p = 0.95 |
|  | **Dust Allergy** | -3.1 | -21.5 | 19.8 | 30 |  |
|  | **No Mould Allergy** | 1.6 | -9.4 | 13.9 | 54 | p = 0.07 |
|  | **Mould Allergy** | **137.2** | **56.5** | **259.6** | **9** |  |
|  | **No Pollen Allergy** | -11.5 | -22.7 | 1.2 | 35 | p = 0.15 |
|  | **Pollen Allergy** | False Convergence | | | |  |
|  | **No Fur Allergy** | -3.3 | -13.2 | 7.7 | 43 | p = 0.17 |
|  | **Fur Allergy** | False Convergence | | | |  |

**Supplemental Information, Table S8.** *Continued.*

| **Ba** | **Exposure**  **Lag Period** | **% Change per IQR** | **Lower 95% CI** | **Upper 95% CI** | **Total Observations** | **Interaction Term** |
| --- | --- | --- | --- | --- | --- | --- |
| **0 day lag** | **All Children** | **8.9** | **2.8** | **15.4** | **169** |  |
|  | **No Allergy** | **9.9** | **0.9** | **19.6** | **52** | p = 0.69 |
|  | **Presence of Any Allergy** | False Convergence | | | |  |
|  | **No Dust Allergy** | **10.4** | **2.8** | **18.6** | **77** | p = 0.27 |
|  | **Dust Allergy** | 6.1 | -2.8 | 15.9 | 92 |  |
|  | **No Mould Allergy** | **7.7** | **1.7** | **14.0** | **133** | p = 0.51 |
|  | **Mould Allergy** | 10.4 | -9.3 | 34.4 | 36 |  |
|  | **No Pollen Allergy** | 2.8 | -3.9 | 10.1 | 102 | p = 0.05 |
|  | **Pollen Allergy** | **18.1** | **5.7** | **32.0** | **67** |  |
|  | **No Fur Allergy** | **8.6** | **2.1** | **15.6** | **100** | p = 0.82 |
|  | **Fur Allergy** | 8.4 | -4.6 | 23.3 | 69 |  |
|  | | | | | |  |
| **1 day lag** | **All Children** | 3.2 | -3.1 | 9.8 | 154 |  |
|  | **No Allergy** | 1.7 | -8.0 | 12.4 | 51 | p = 0.97 |
|  | **Presence of Any Allergy** | 2.4 | -6.4 | 12.1 | 103 |  |
|  | **No Dust Allergy** | 6.4 | -2.4 | 16.1 | 73 | p = 0.10 |
|  | **Dust Allergy** | -3.1 | -11.1 | 5.6 | 81 |  |
|  | **No Mould Allergy** | 2.0 | -4.6 | 9.0 | 125 | p = 0.50 |
|  | **Mould Allergy** | 9.9 | -10.9 | 35.7 | 29 |  |
|  | **No Pollen Allergy** | 0.0 | -6.8 | 7.2 | 91 | p = 0.34 |
|  | **Pollen Allergy** | 8.6 | -2.0 | 20.3 | 63 |  |
|  | **No Fur Allergy** | 0.7 | -6.5 | 8.4 | 94 | p = 0.11 |
|  | **Fur Allergy** | 8.7 | -4.3 | 23.3 | 60 |  |
|  | | | | | |  |
| **2 day lag** | **All Children** | 4.6 | -2.8 | 12.5 | 142 |  |
|  | **No Allergy** | False Convergence | | | | p = 0.29 |
|  | **Presence of Any Allergy** | 1.4 | -7.4 | 11.0 | 99 |  |
|  | **No Dust Allergy** | 6.1 | -7.8 | 22.0 | 62 | p = 0.95 |
|  | **Dust Allergy** | 5.8 | -2.0 | 14.2 | 80 |  |
|  | **No Mould Allergy** | 4.9 | -3.8 | 14.4 | 113 | p = 0.79 |
|  | **Mould Allergy** | 8.7 | -10.6 | 32.0 | 29 |  |
|  | **No Pollen Allergy** | 3.8 | -4.3 | 12.5 | 82 | p = 0.86 |
|  | **Pollen Allergy** | 5.0 | -5.4 | 16.4 | 60 |  |
|  | **No Fur Allergy** | 2.1 | -5.9 | 10.8 | 84 | p = 0.16 |
|  | **Fur Allergy** | 10.2 | -1.9 | 23.8 | 58 |  |

**Supplemental Information, Table S8.** *Continued.*

| **Cr** | **Exposure**  **Lag Period** | **% Change per IQR** | **Lower 95% CI** | **Upper 95% CI** | **Total Observations** | **Interaction Term** |
| --- | --- | --- | --- | --- | --- | --- |
| **0 day lag** | **All Children** | 1.8 | -0.3 | 4.0 | 184 |  |
|  | **No Allergy** | **7.7** | **0.3** | **15.6** | **51** | p = 0.15 |
|  | **Presence of Any Allergy** | False Convergence | | | |  |
|  | **No Dust Allergy** | **5.8** | **0.5** | **11.4** | **80** | p = 0.06 |
|  | **Dust Allergy** | 1.3 | -1.1 | 3.8 | 104 |  |
|  | **No Mould Allergy** | **2.4** | **0.1** | **4.8** | **144** | p = 0.15 |
|  | **Mould Allergy** | -4.1 | -13.4 | 6.3 | 40 |  |
|  | **No Pollen Allergy** | 1.4 | -1.4 | 4.3 | 116 | p = 0.77 |
|  | **Pollen Allergy** | 2.1 | -1.4 | 5.7 | 68 |  |
|  | **No Fur Allergy** | 3.2 | -0.1 | 6.7 | 113 | p = 0.34 |
|  | **Fur Allergy** | 0.6 | -2.1 | 3.4 | 71 |  |
|  | | | | | |  |
| **1 day lag** | **All Children** | 0.3 | -1.8 | 2.6 | 163 |  |
|  | **No Allergy** | 1.8 | -5.7 | 9.8 | 48 | p = 0.90 |
|  | **Presence of Any Allergy** | 0.2 | -1.6 | 2.2 | 115 |  |
|  | **No Dust Allergy** | 2.1 | -3.7 | 8.2 | 73 | p = 0.67 |
|  | **Dust Allergy** | 0.0 | -1.8 | 1.9 | 90 |  |
|  | **No Mould Allergy** | 0.4 | -2.1 | 2.9 | 132 | p = 0.90 |
|  | **Mould Allergy** | -0.6 | -6.9 | 6.1 | 31 |  |
|  | **No Pollen Allergy** | 0.0 | -2.7 | 2.9 | 101 | p = 0.90 |
|  | **Pollen Allergy** | 1.0 | -2.5 | 4.6 | 62 |  |
|  | **No Fur Allergy** | 0.6 | -2.7 | 3.9 | 102 | p = 0.77 |
|  | **Fur Allergy** | 0.5 | -2.0 | 3.1 | 61 |  |
|  | | | | | |  |
| **2 day lag** | **All Children** | 0.7 | -1.2 | 2.7 | 146 |  |
|  | **No Allergy** | False Convergence | | | | p = 0.73 |
|  | **Presence of Any Allergy** | 0.5 | -1.3 | 2.4 | 101 |  |
|  | **No Dust Allergy** | 2.8 | -2.3 | 8.3 | 66 | p = 0.27 |
|  | **Dust Allergy** | 0.1 | -1.7 | 2.0 | 80 |  |
|  | **No Mould Allergy** | 0.9 | -1.2 | 3.2 | 118 | p = 0.25 |
|  | **Mould Allergy** | -1.3 | -9.1 | 7.2 | 28 |  |
|  | **No Pollen Allergy** | 1.4 | -0.8 | 3.6 | 88 | p = 0.44 |
|  | **Pollen Allergy** | 0.5 | -3.0 | 4.1 | 58 |  |
|  | **No Fur Allergy** | 0.5 | -2.2 | 3.4 | 92 | p = 0.91 |
|  | **Fur Allergy** | 0.5 | -1.7 | 2.8 | 54 |  |

**Supplemental Information, Table S8.** *Continued.*

| **Cu** | **Exposure**  **Lag Period** | **% Change per IQR** | **Lower 95% CI** | **Upper 95% CI** | **Total Observations** | **Interaction Term** |
| --- | --- | --- | --- | --- | --- | --- |
| **0 day lag** | **All Children** | 1.2 | -1.4 | 4.0 | 207 |  |
|  | **No Allergy** | False Convergence | | | | p = 0.11 |
|  | **Presence of Any Allergy** | 1.0 | -1.1 | 3.2 | 59 |  |
|  | **No Dust Allergy** | 0.5 | -4.7 | 6.0 | 94 | p = 0.79 |
|  | **Dust Allergy** | 0.7 | -2.6 | 4.1 | 113 |  |
|  | **No Mould Allergy** | 1.2 | -2.4 | 4.8 | 165 | p = 0.63 |
|  | **Mould Allergy** | 0.9 | -2.4 | 4.4 | 42 |  |
|  | **No Pollen Allergy** | False Convergence | | | | p = 0.14 |
|  | **Pollen Allergy** | 1.7 | -0.8 | 4.2 | 78 |  |
|  | **No Fur Allergy** | 0.4 | -3.8 | 4.9 | 121 | p = 0.31 |
|  | **Fur Allergy** | 2.9 | -0.9 | 6.8 | 86 |  |
|  | | | | | |  |
| **1 day lag** | **All Children** | 2.6 | -1.0 | 6.2 | 188 |  |
|  | **No Allergy** | 0.8 | -5.8 | 7.8 | 58 | p = 0.95 |
|  | **Presence of Any Allergy** | 2.8 | -1.5 | 7.2 | 130 |  |
|  | **No Dust Allergy** | -0.1 | -4.8 | 4.7 | 89 | p = 0.21 |
|  | **Dust Allergy** | 4.8 | -0.4 | 10.1 | 99 |  |
|  | **No Mould Allergy** | 2.4 | -1.7 | 6.7 | 155 | p = 0.20 |
|  | **Mould Allergy** | **11.7** | **2.7** | **21.4** | **33** |  |
|  | **No Pollen Allergy** | 1.0 | -4.1 | 6.4 | 117 | p = 0.42 |
|  | **Pollen Allergy** | 2.0 | -1.0 | 5.2 | 71 |  |
|  | **No Fur Allergy** | 2.6 | -1.6 | 6.9 | 114 | p = 0.84 |
|  | **Fur Allergy** | 3.5 | -4.1 | 11.7 | 74 |  |
|  | | | | | |  |
| **2 day lag** | **All Children** | 0.1 | -3.5 | 3.9 | 168 |  |
|  | **No Allergy** | 0.1 | -2.7 | 3.0 | 52 | **p = 0.04** |
|  | **Presence of Any Allergy** | -3.0 | -7.5 | 1.8 | 116 |  |
|  | **No Dust Allergy** | -2.3 | -7.9 | 3.7 | 79 | p = 0.16 |
|  | **Dust Allergy** | 1.4 | -3.9 | 7.0 | 89 |  |
|  | **No Mould Allergy** | -0.7 | -5.1 | 3.9 | 137 | **p = 0.003** |
|  | **Mould Allergy** | False Convergence | | | |  |
|  | **No Pollen Allergy** | 4.6 | -1.1 | 10.7 | 101 | p = 0.07 |
|  | **Pollen Allergy** | False Convergence | | | |  |
|  | **No Fur Allergy** | 4.2 | -0.3 | 9.0 | 100 | **p = 0.01** |
|  | **Fur Allergy** | False Convergence | | | |  |

**Supplemental Information, Table S8.** *Continued.*

| **Fe** | **Exposure**  **Lag Period** | **% Change per IQR** | **Lower 95% CI** | **Upper 95% CI** | **Total Observations** | **Interaction Term** |
| --- | --- | --- | --- | --- | --- | --- |
| **0 day lag** | **All Children** | 0.8 | -3.8 | 5.7 | 212 |  |
|  | **No Allergy** | 0.7 | -3.1 | 4.6 | 61 | p = 0.25 |
|  | **Presence of Any Allergy** | False Convergence | | | |  |
|  | **No Dust Allergy** | 2.2 | -6.8 | 12.1 | 97 | p = 0.51 |
|  | **Dust Allergy** | -1.1 | -5.5 | 3.4 | 115 |  |
|  | **No Mould Allergy** | 1.0 | -4.4 | 6.8 | 172 | p = 0.90 |
|  | **Mould Allergy** | -3.4 | -11.6 | 5.6 | 40 |  |
|  | **No Pollen Allergy** | 1.4 | -4.4 | 7.5 | 131 | p = 0.79 |
|  | **Pollen Allergy** | -0.6 | -9.0 | 8.5 | 81 |  |
|  | **No Fur Allergy** | 4.4 | -2.4 | 11.8 | 127 | p = 0.16 |
|  | **Fur Allergy** | -2.6 | -9.0 | 4.3 | 85 |  |
|  | | | | | |  |
| **1 day lag** | **All Children** | **7.5** | **1.5** | **13.9** | **191** |  |
|  | **No Allergy** | 10.4 | -1.1 | 23.2 | 58 | p = 0.19 |
|  | **Presence of Any Allergy** | 5.0 | -1.5 | 11.8 | 133 |  |
|  | **No Dust Allergy** | **16.0** | **3.6** | **29.9** | **90** | **p = 0.01** |
|  | **Dust Allergy** | 1.4 | -3.8 | 7.0 | 101 |  |
|  | **No Mould Allergy** | **7.9** | **1.2** | **15.2** | **159** | p = 0.94 |
|  | **Mould Allergy** | 10.7 | -3.9 | 27.6 | 32 |  |
|  | **No Pollen Allergy** | **9.7** | **2.7** | **17.3** | **117** | p = 0.36 |
|  | **Pollen Allergy** | 7.7 | -4.5 | 21.4 | 74 |  |
|  | **No Fur Allergy** | **7.7** | **0.3** | **15.6** | **117** | p = 0.84 |
|  | **Fur Allergy** | 8.2 | -2.1 | 19.6 | 74 |  |
|  | | | | | |  |
| **2 day lag** | **All Children** | 3.0 | -2.9 | 9.2 | 173 |  |
|  | **No Allergy** | False Convergence | | | | **p = 0.01** |
|  | **Presence of Any Allergy** | -1.7 | -7.8 | 4.8 | 120 |  |
|  | **No Dust Allergy** | 8.0 | -5.6 | 23.5 | 81 | p = 0.19 |
|  | **Dust Allergy** | -0.4 | -5.3 | 4.6 | 92 |  |
|  | **No Mould Allergy** | 2.0 | -4.5 | 8.9 | 142 | p = 0.35 |
|  | **Mould Allergy** | 4.1 | -11.7 | 22.6 | 31 |  |
|  | **No Pollen Allergy** | **6.5** | **0.0** | **13.5** | **103** | p = 0.18 |
|  | **Pollen Allergy** | -0.8 | -12.0 | 11.8 | 70 |  |
|  | **No Fur Allergy** | 5.0 | -1.6 | 12.0 | 104 | p = 0.27 |
|  | **Fur Allergy** | -2.2 | -13.1 | 10.1 | 69 |  |

**Supplemental Information, Table S8.** *Continued.*

| **Mg** | **Exposure**  **Lag Period** | **% Change per IQR** | **Lower 95% CI** | **Upper 95% CI** | **Total Observations** | **Interaction Term** |
| --- | --- | --- | --- | --- | --- | --- |
| **0 day lag** | **All Children** | 2.3 | -3.3 | 8.2 | 179 |  |
|  | **No Allergy** | 4.7 | -8.5 | 19.9 | 53 | p = 0.71 |
|  | **Presence of Any Allergy** | False Convergence | | | |  |
|  | **No Dust Allergy** | **11.0** | **0.3** | **22.9** | **84** | **p = 0.03** |
|  | **Dust Allergy** | -2.5 | -7.6 | 3.0 | 95 |  |
|  | **No Mould Allergy** | 2.4 | -4.5 | 9.9 | 142 | p = 0.97 |
|  | **Mould Allergy** | 3.9 | -6.9 | 15.9 | 37 |  |
|  | **No Pollen Allergy** | 4.1 | -3.4 | 12.3 | 109 | p = 0.67 |
|  | **Pollen Allergy** | -0.4 | -8.7 | 8.8 | 70 |  |
|  | **No Fur Allergy** | 3.1 | -5.1 | 12.1 | 107 | p = 0.94 |
|  | **Fur Allergy** | 1.6 | -4.9 | 8.5 | 72 |  |
|  | | | | | |  |
| **1 day lag** | **All Children** | **7.7** | **1.8** | **13.9** | **163** |  |
|  | **No Allergy** | 9.5 | -2.7 | 23.3 | 51 | p = 0.62 |
|  | **Presence of Any Allergy** | **6.6** | **0.3** | **13.2** | **112** |  |
|  | **No Dust Allergy** | **13.2** | **1.3** | **26.6** | **80** | p = 0.08 |
|  | **Dust Allergy** | 5.0 | -0.6 | 10.9 | 83 |  |
|  | **No Mould Allergy** | 5.6 | -1.1 | 12.7 | 133 | p = 0.85 |
|  | **Mould Allergy** | **13.4** | **2.1** | **26.1** | **30** |  |
|  | **No Pollen Allergy** | 7.4 | -0.4 | 15.8 | 98 | p = 0.57 |
|  | **Pollen Allergy** | 7.4 | -1.5 | 17.2 | 65 |  |
|  | **No Fur Allergy** | 6.8 | -1.4 | 15.8 | 100 | p = 0.81 |
|  | **Fur Allergy** | 6.0 | -0.7 | 13.1 | 63 |  |
|  | | | | | |  |
| **2 day lag** | **All Children** | 5.6 | -0.5 | 12.0 | 146 |  |
|  | **No Allergy** | False Convergence | | | | p = 0.06 |
|  | **Presence of Any Allergy** | 0.8 | -5.1 | 7.2 | 99 |  |
|  | **No Dust Allergy** | **16.8** | **3.4** | **32.1** | **71** | **p = 0.02** |
|  | **Dust Allergy** | 0.4 | -4.7 | 5.8 | 75 |  |
|  | **No Mould Allergy** | 5.7 | -1.5 | 13.3 | 117 | p = 0.84 |
|  | **Mould Allergy** | 4.6 | -4.2 | 14.2 | 29 |  |
|  | **No Pollen Allergy** | 7.4 | -0.5 | 15.9 | 84 | p = 0.92 |
|  | **Pollen Allergy** | 4.5 | -5.2 | 15.2 | 62 |  |
|  | **No Fur Allergy** | 6.8 | -2.0 | 16.5 | 87 | p = 0.71 |
|  | **Fur Allergy** | False Convergence | | | |  |

**Supplemental Information, Table S8.** *Continued.*

| **Mn** | **Exposure**  **Lag Period** | **% Change per IQR** | **Lower 95% CI** | **Upper 95% CI** | **Total Observations** | **Interaction Term** |
| --- | --- | --- | --- | --- | --- | --- |
| **0 day lag** | **All Children** | -1.2 | -4.7 | 2.4 | 204 |  |
|  | **No Allergy** | False Convergence | | | | p = 0.71 |
|  | **Presence of Any Allergy** | False Convergence | | | |  |
|  | **No Dust Allergy** | -1.2 | -5.3 | 3.2 | 93 | p = 0.73 |
|  | **Dust Allergy** | -1.5 | -6.5 | 3.7 | 111 |  |
|  | **No Mould Allergy** | -1.2 | -5.0 | 2.8 | 165 | p = 0.92 |
|  | **Mould Allergy** | False Convergence | | | |  |
|  | **No Pollen Allergy** | -0.3 | -3.6 | 3.1 | 127 | p = 0.57 |
|  | **Pollen Allergy** | -4.5 | -13.3 | 5.3 | 77 |  |
|  | **No Fur Allergy** | -0.3 | -3.8 | 3.3 | 122 | p = 0.69 |
|  | **Fur Allergy** | -3.5 | -10.2 | 3.7 | 82 |  |
|  | | | | | |  |
| **1 day lag** | **All Children** | -0.5 | -4.7 | 3.9 | 182 |  |
|  | **No Allergy** | 0.4 | -3.8 | 4.8 | 56 | p = 0.40 |
|  | **Presence of Any Allergy** | -3.0 | -9.6 | 4.0 | 126 |  |
|  | **No Dust Allergy** | -0.2 | -5.5 | 5.4 | 86 | p = 0.46 |
|  | **Dust Allergy** | -3.7 | -10.6 | 3.8 | 96 |  |
|  | **No Mould Allergy** | -0.1 | -4.5 | 4.5 | 152 | p = 0.31 |
|  | **Mould Allergy** | -14.0 | -32.1 | 8.9 | 30 |  |
|  | **No Pollen Allergy** | 0.1 | -3.7 | 4.1 | 113 | p = 0.74 |
|  | **Pollen Allergy** | -3.0 | -16.4 | 12.6 | 69 |  |
|  | **No Fur Allergy** | -0.4 | -4.1 | 3.5 | 113 | p = 0.97 |
|  | **Fur Allergy** | -1.6 | -12.8 | 10.9 | 69 |  |
|  | | | | | |  |
| **2 day lag** | **All Children** | 2.4 | -2.7 | 7.7 | 164 |  |
|  | **No Allergy** | False Convergence | | | | p = 0.79 |
|  | **Presence of Any Allergy** | 1.4 | -4.6 | 7.7 | 114 |  |
|  | **No Dust Allergy** | 3.7 | -4.9 | 13.0 | 76 | p = 0.53 |
|  | **Dust Allergy** | 0.3 | -5.2 | 6.1 | 88 |  |
|  | **No Mould Allergy** | 1.8 | -3.7 | 7.7 | 135 | p = 0.49 |
|  | **Mould Allergy** | 1.2 | -13.3 | 18.2 | 29 |  |
|  | **No Pollen Allergy** | 3.8 | -1.2 | 8.9 | 98 | p = 0.47 |
|  | **Pollen Allergy** | False Convergence | | | |  |
|  | **No Fur Allergy** | 2.8 | -1.9 | 7.8 | 99 | p = 0.78 |
|  | **Fur Allergy** | -0.7 | -10.5 | 10.1 | 65 |  |

**Supplemental Information, Table S8.** *Continued.*

| **Ni** | **Exposure**  **Lag Period** | **% Change per IQR** | **Lower 95% CI** | **Upper 95% CI** | **Total Observations** | **Interaction Term** |
| --- | --- | --- | --- | --- | --- | --- |
| **0 day lag** | **All Children** | 1.1 | -2.7 | 5.1 | 173 |  |
|  | **No Allergy** | 3.2 | -5.2 | 12.4 | 50 | p = 0.84 |
|  | **Presence of Any Allergy** | False Convergence | | | |  |
|  | **No Dust Allergy** | 2.3 | -4.0 | 9.1 | 78 | p = 0.70 |
|  | **Dust Allergy** | -0.4 | -4.9 | 4.3 | 95 |  |
|  | **No Mould Allergy** | 1.6 | -2.5 | 6.0 | 139 | p = 0.54 |
|  | **Mould Allergy** | -5.7 | -16.3 | 6.2 | 34 |  |
|  | **No Pollen Allergy** | 3.0 | -1.7 | 7.9 | 107 | p = 0.26 |
|  | **Pollen Allergy** | -1.4 | -7.7 | 5.2 | 66 |  |
|  | **No Fur Allergy** | 3.1 | -1.4 | 7.8 | 104 | p = 0.09 |
|  | **Fur Allergy** | -4.8 | -12.0 | 3.1 | 69 |  |
|  | | | | | |  |
| **1 day lag** | **All Children** | 1.5 | -2.8 | 6.1 | 153 |  |
|  | **No Allergy** | 3.8 | -8.5 | 17.6 | 47 | p = 0.77 |
|  | **Presence of Any Allergy** | 0.6 | -3.7 | 5.2 | 106 |  |
|  | **No Dust Allergy** | 2.6 | -6.0 | 12.1 | 71 | p = 0.88 |
|  | **Dust Allergy** | 0.4 | -3.7 | 4.7 | 82 |  |
|  | **No Mould Allergy** | 1.6 | -2.9 | 6.3 | 128 | p = 0.80 |
|  | **Mould Allergy** | -11.5 | -36.0 | 22.5 | 25 |  |
|  | **No Pollen Allergy** | 7.3 | 0.4 | 14.6 | 95 | **p = 0.05** |
|  | **Pollen Allergy** | -1.9 | -7.4 | 4.0 | 58 |  |
|  | **No Fur Allergy** | 3.1 | -1.8 | 8.3 | 95 | p = 0.17 |
|  | **Fur Allergy** | -4.9 | -15.2 | 6.6 | 58 |  |
|  | | | | | |  |
| **2 day lag** | **All Children** | 2.4 | -1.9 | 6.8 | 138 |  |
|  | **No Allergy** | False Convergence | | | | p = 0.70 |
|  | **Presence of Any Allergy** | 2.4 | -1.9 | 6.8 | 95 |  |
|  | **No Dust Allergy** | 5.0 | -5.6 | 16.7 | 63 | p = 0.71 |
|  | **Dust Allergy** | 1.3 | -2.5 | 5.4 | 75 |  |
|  | **No Mould Allergy** | 2.0 | -2.7 | 7.0 | 113 | p = 0.55 |
|  | **Mould Allergy** | **20.5** | **0.1** | **45.1** | **25** |  |
|  | **No Pollen Allergy** | **10.2** | **4.5** | **16.3** | **84** | **p = 0.01** |
|  | **Pollen Allergy** | -2.6 | -9.2 | 4.4 | 54 |  |
|  | **No Fur Allergy** | **5.2** | **0.1** | **10.5** | **85** | **p = 0.03** |
|  | **Fur Allergy** | -10.0 | -20.0 | 1.3 | 53 |  |

**Supplemental Information, Table S8.** *Continued.*

| **S** | **Exposure**  **Lag Period** | **% Change per IQR** | **Lower 95% CI** | **Upper 95% CI** | **Total Observations** | **Interaction Term** |
| --- | --- | --- | --- | --- | --- | --- |
| **0 day lag** | **All Children** | 3.1 | -1.7 | 8.2 | 214 |  |
|  | **No Allergy** | False Convergence | | | | p = 0.59 |
|  | **Presence of Any Allergy** | 3.1 | -1.8 | 8.2 | 61 |  |
|  | **No Dust Allergy** | 1.6 | -6.3 | 10.2 | 97 | p = 0.55 |
|  | **Dust Allergy** | 3.0 | -2.5 | 8.7 | 117 |  |
|  | **No Mould Allergy** | 2.9 | -2.3 | 8.3 | 172 | p = 0.67 |
|  | **Mould Allergy** | -3.7 | -17.2 | 11.9 | 42 |  |
|  | **No Pollen Allergy** | 2.9 | -1.8 | 7.8 | 133 | p = 0.92 |
|  | **Pollen Allergy** | 1.0 | -13.0 | 17.3 | 81 |  |
|  | **No Fur Allergy** | 3.3 | -2.3 | 9.3 | 127 | p = 0.82 |
|  | **Fur Allergy** | 0.2 | -10.3 | 12.0 | 87 |  |
|  | | | | | |  |
| **1 day lag** | **All Children** | 2.5 | -2.9 | 8.2 | 192 |  |
|  | **No Allergy** | 3.8 | -6.1 | 14.7 | 58 | p = 0.50 |
|  | **Presence of Any Allergy** | 1.0 | -4.6 | 7.1 | 134 |  |
|  | **No Dust Allergy** | 3.2 | -5.0 | 12.2 | 90 | p = 0.39 |
|  | **Dust Allergy** | -0.2 | -6.2 | 6.3 | 102 |  |
|  | **No Mould Allergy** | 2.6 | -3.2 | 8.6 | 159 | p = 0.84 |
|  | **Mould Allergy** | -2.9 | -18.7 | 15.9 | 33 |  |
|  | **No Pollen Allergy** | 3.4 | -1.4 | 8.5 | 118 | p = 0.52 |
|  | **Pollen Allergy** | 1.3 | -13.9 | 19.2 | 74 |  |
|  | **No Fur Allergy** | 1.3 | -4.9 | 8.0 | 117 | p = 0.84 |
|  | **Fur Allergy** | 0.8 | -10.2 | 13.1 | 75 |  |
|  | | | | | |  |
| **2 day lag** | **All Children** | 3.3 | -2.2 | 9.1 | 173 |  |
|  | **No Allergy** | False Convergence | | | | p = 0.15 |
|  | **Presence of Any Allergy** | 0.4 | -5.4 | 6.5 | 120 |  |
|  | **No Dust Allergy** | 6.5 | -3.1 | 17.0 | 81 | p = 0.21 |
|  | **Dust Allergy** | -2.0 | -8.1 | 4.4 | 92 |  |
|  | **No Mould Allergy** | 2.4 | -3.5 | 8.8 | 142 | p = 0.16 |
|  | **Mould Allergy** | 6.0 | -12.6 | 28.6 | 31 |  |
|  | **No Pollen Allergy** | 3.7 | -0.7 | 8.4 | 103 | p = 0.57 |
|  | **Pollen Allergy** | 1.2 | -14.2 | 19.3 | 70 |  |
|  | **No Fur Allergy** | 2.8 | -2.7 | 8.7 | 104 | p = 0.90 |
|  | **Fur Allergy** | 1.0 | -9.7 | 13.0 | 69 |  |

**Supplemental Information, Table S8.** *Continued.*

| **Sb** | **Exposure**  **Lag Period** | **% Change per IQR** | **Lower 95% CI** | **Upper 95% CI** | **Total Observations** | **Interaction Term** |
| --- | --- | --- | --- | --- | --- | --- |
| **0 day lag** | **All Children** | 1.7 | -14.7 | 21.3 | 57 |  |
|  | **No Allergy** | False Convergence | | | | False Convergence |
|  | **Presence of Any Allergy** | 2.0 | -16.8 | 25.2 | 14 |  |
|  | **No Dust Allergy** | 35.2 | 1.5 | 80.1 | 25 | False Convergence |
|  | **Dust Allergy** | -2.9 | -23.6 | 23.5 | 32 |  |
|  | **No Mould Allergy** | -9.7 | -27.7 | 12.7 | 45 | p = 0.42 |
|  | **Mould Allergy** | **14.9** | **3.6** | **27.5** | **12** |  |
|  | **No Pollen Allergy** | **39.2** | **8.9** | **77.9** | **37** | **p = 0.003** |
|  | **Pollen Allergy** | False Convergence | | | |  |
|  | **No Fur Allergy** | 16.3 | -12.0 | 53.8 | 35 | p = 0.36 |
|  | **Fur Allergy** | -10.1 | -28.7 | 13.3 | 22 |  |
|  | | | | | |  |
| **1 day lag** | **All Children** | False Convergence | | | |  |
|  | **No Allergy** | False Convergence | | | | False Convergence |
|  | **Presence of Any Allergy** | False Convergence | | | |  |
|  | **No Dust Allergy** | False Convergence | | | | False Convergence |
|  | **Dust Allergy** | 9.6 | -6.7 | 28.8 | 31 |  |
|  | **No Mould Allergy** | False Convergence | | | | False Convergence |
|  | **Mould Allergy** | **140.2** | **118.0** | **164.6** | **10** |  |
|  | **No Pollen Allergy** | **33.1** | **11.3** | **59.1** | **36** | **p = 0.02** |
|  | **Pollen Allergy** | -42.1 | -65.5 | -2.7 | 20 |  |
|  | **No Fur Allergy** | **31.2** | **8.5** | **58.7** | **37** | False Convergence |
|  | **Fur Allergy** | False Convergence | | | |  |
|  | | | | | |  |
| **2 day lag** | **All Children** | 2.7 | -12.8 | 20.9 | 50 |  |
|  | **No Allergy** | -15.4 |  |  | 13 | p = 0.89 |
|  | **Presence of Any Allergy** | 1.7 | -16.3 | 23.4 | 37 |  |
|  | **No Dust Allergy** | 2.3 | -21.5 | 33.2 | 23 | p = 0.16 |
|  | **Dust Allergy** | 4.7 | -15.6 | 29.9 | 27 |  |
|  | **No Mould Allergy** | 1.3 | -14.7 | 20.4 | 42 | p = 0.61 |
|  | **Mould Allergy** | 0.5 | -5.9 | 7.4 | 8 |  |
|  | **No Pollen Allergy** | 9.0 | -20.4 | 49.3 | 31 | p = 0.32 |
|  | **Pollen Allergy** | False Convergence | | | |  |
|  | **No Fur Allergy** | 16.4 | -11.6 | 53.3 | 32 | p = 0.56 |
|  | **Fur Allergy** | False Convergence | | | |  |

**Supplemental Information, Table S8.** *Continued.*

| **Sr** | **Exposure**  **Lag Period** | **% Change per IQR** | **Lower 95% CI** | **Upper 95% CI** | **Total Observations** | **Interaction Term** |
| --- | --- | --- | --- | --- | --- | --- |
| **0 day lag** | **All Children** | 1.4 | -3.5 | 6.6 | 107 |  |
|  | **No Allergy** | False Convergence | | | | False Convergence |
|  | **Presence of Any Allergy** | False Convergence | | | |  |
|  | **No Dust Allergy** | 2.5 | -8.2 | 14.3 | 40 | p = 0.69 |
|  | **Dust Allergy** | False Convergence | | | |  |
|  | **No Mould Allergy** | 0.3 | -4.9 | 5.9 | 89 | p = 0.76 |
|  | **Mould Allergy** | False Convergence | | | |  |
|  | **No Pollen Allergy** | -3.1 | -10.4 | 4.7 | 70 | p = 0.14 |
|  | **Pollen Allergy** | 6.3 | -5.4 | 19.5 | 37 |  |
|  | **No Fur Allergy** | -3.2 | -9.2 | 3.2 | 63 | **p = 0.04** |
|  | **Fur Allergy** | False Convergence | | | |  |
|  | | | | | |  |
| **1 day lag** | **All Children** | -1.5 | -6.2 | 3.4 | 97 |  |
|  | **No Allergy** | -3.3 | -15.5 | 10.7 | 26 | p = 0.64 |
|  | **Presence of Any Allergy** | -2.3 | -6.9 | 2.5 | 71 |  |
|  | **No Dust Allergy** | 1.5 | -7.3 | 11.1 | 37 | p = 0.52 |
|  | **Dust Allergy** | 1.0 | -4.2 | 6.4 | 60 |  |
|  | **No Mould Allergy** | -1.1 | -6.1 | 4.1 | 84 | p = 0.82 |
|  | **Mould Allergy** | False Convergence | | | |  |
|  | **No Pollen Allergy** | -5.0 | -12.2 | 2.7 | 63 | p = 0.34 |
|  | **Pollen Allergy** | -2.1 | -11.5 | 8.4 | 34 |  |
|  | **No Fur Allergy** | -1.3 | -6.9 | 4.7 | 60 | p = 0.99 |
|  | **Fur Allergy** | -1.2 | -12.8 | 11.9 | 37 |  |
|  | | | | | |  |
| **2 day lag** | **All Children** | 1.4 | -1.8 | 4.6 | 88 |  |
|  | **No Allergy** | 0.9 | -8.4 | 11.1 | 24 | False Convergence |
|  | **Presence of Any Allergy** | 0.9 | -3.1 | 5.2 | 64 |  |
|  | **No Dust Allergy** | False Convergence | | | | False Convergence |
|  | **Dust Allergy** | 0.5 | -3.2 | 4.3 | 55 |  |
|  | **No Mould Allergy** | False Convergence | | | | False Convergence |
|  | **Mould Allergy** | False Convergence | | | |  |
|  | **No Pollen Allergy** | 2.2 | -3.1 | 7.7 | 53 | False Convergence |
|  | **Pollen Allergy** | False Convergence | | | |  |
|  | **No Fur Allergy** | 2.1 | -1.6 | 5.9 | 53 | False Convergence |
|  | **Fur Allergy** | False Convergence | | | |  |

**Supplemental Information, Table S8.** *Continued.*

| **V** | **Exposure**  **Lag Period** | **% Change per IQR** | **Lower 95% CI** | **Upper 95% CI** | **Total Observations** | **Interaction Term** |
| --- | --- | --- | --- | --- | --- | --- |
| **0 day lag** | **All Children** | 5.0 | -2.9 | 13.5 | 130 |  |
|  | **No Allergy** | 8.3 | -11.9 | 33.1 | 43 | p = 0.91 |
|  | **Presence of Any Allergy** | False Convergence | | | |  |
|  | **No Dust Allergy** | 4.9 | -6.4 | 17.5 | 61 | p = 0.98 |
|  | **Dust Allergy** | 3.9 | -5.0 | 13.5 | 69 |  |
|  | **No Mould Allergy** | 3.4 | -4.7 | 12.3 | 107 | p = 0.84 |
|  | **Mould Allergy** | False Convergence | | | |  |
|  | **No Pollen Allergy** | False Convergence | | | | p = 0.29 |
|  | **Pollen Allergy** | 8.2 | -11.3 | 31.9 | 48 |  |
|  | **No Fur Allergy** | 1.2 | -8.1 | 11.4 | 81 | p = 0.34 |
|  | **Fur Allergy** | False Convergence | | | |  |
|  | | | | | |  |
| **1 day lag** | **All Children** | 0.8 | -6.1 | 8.1 | 120 |  |
|  | **No Allergy** | -0.6 | -13.6 | 14.4 | 42 | p = 0.85 |
|  | **Presence of Any Allergy** | -0.1 | -7.5 | 7.8 | 78 |  |
|  | **No Dust Allergy** | -0.7 | -10.4 | 10.0 | 56 | p = 0.92 |
|  | **Dust Allergy** | False Convergence | | | |  |
|  | **No Mould Allergy** | 0.0 | -6.3 | 6.8 | 100 | p = 0.69 |
|  | **Mould Allergy** | 40.9 | -9.5 | 119.3 | 20 |  |
|  | **No Pollen Allergy** | -1.1 | -7.1 | 5.3 | 74 | p = 0.18 |
|  | **Pollen Allergy** | 11.2 | -10.1 | 37.5 | 46 |  |
|  | **No Fur Allergy** | -0.2 | -7.2 | 7.4 | 76 | p = 0.68 |
|  | **Fur Allergy** | False Convergence | | | |  |
|  | | | | | |  |
| **2 day lag** | **All Children** | **7.6** | **0.1** | **15.8** | **110** |  |
|  | **No Allergy** | False Convergence | | | | p = 0.44 |
|  | **Presence of Any Allergy** | 5.1 | -3.0 | 13.9 | 73 |  |
|  | **No Dust Allergy** | 10.7 | -0.3 | 22.9 | 51 | p = 0.25 |
|  | **Dust Allergy** | 3.0 | -4.5 | 11.1 | 59 |  |
|  | **No Mould Allergy** | **32.2** | **12.9** | **54.9** | **18** | p = 0.96 |
|  | **Mould Allergy** | 3.3 | -14.1 | 24.2 | 46 |  |
|  | **No Pollen Allergy** | **7.9** | **2.2** | **14.0** | **64** | p = 0.69 |
|  | **Pollen Allergy** | 6.2 | -0.7 | 13.5 | 92 |  |
|  | **No Fur Allergy** | 7.3 | -0.4 | 15.5 | 67 | p = 0.87 |
|  | **Fur Allergy** | 6.6 | -10.7 | 27.1 | 43 |  |

**Supplemental Information, Table S8.** *Continued.*

| **Zn** | **Exposure**  **Lag Period** | **% Change per IQR** | **Lower 95% CI** | **Upper 95% CI** | **Total Observations** | **Interaction Term** |
| --- | --- | --- | --- | --- | --- | --- |
| **0 day lag** | **All Children** | -0.9 | -4.3 | 2.5 | 208 |  |
|  | **No Allergy** | False Convergence | | | | p = 0.48 |
|  | **Presence of Any Allergy** | False Convergence | | | |  |
|  | **No Dust Allergy** | -2.3 | -6.5 | 2.0 | 96 | p = 0.44 |
|  | **Dust Allergy** | False Convergence | | | |  |
|  | **No Mould Allergy** | -0.8 | -3.9 | 2.5 | 168 | p = 0.90 |
|  | **Mould Allergy** | False Convergence | | | |  |
|  | **No Pollen Allergy** | False Convergence | | | | p = 0.99 |
|  | **Pollen Allergy** | -0.9 | -5.6 | 4.0 | 78 |  |
|  | **No Fur Allergy** | 0.3 | -4.1 | 4.8 | 124 | p = 0.51 |
|  | **Fur Allergy** | -2.5 | -7.3 | 2.5 | 84 |  |
|  | | | | | |  |
| **1 day lag** | **All Children** | 1.7 | -1.8 | 5.2 | 188 |  |
|  | **No Allergy** | 0.2 | -4.7 | 5.4 | 58 | p = 0.72 |
|  | **Presence of Any Allergy** | 2.3 | -2.1 | 6.8 | 130 |  |
|  | **No Dust Allergy** | -0.9 | -5.2 | 3.6 | 89 | p = 0.43 |
|  | **Dust Allergy** | 2.2 | -2.1 | 6.7 | 99 |  |
|  | **No Mould Allergy** | 1.4 | -1.8 | 4.6 | 156 | p = 0.41 |
|  | **Mould Allergy** | 8.2 | -12.1 | 33.1 | 32 |  |
|  | **No Pollen Allergy** | 0.8 | -3.1 | 4.8 | 116 | p = 0.54 |
|  | **Pollen Allergy** | 3.0 | -2.7 | 9.1 | 72 |  |
|  | **No Fur Allergy** | 0.6 | -3.7 | 5.1 | 115 | p = 0.57 |
|  | **Fur Allergy** | 2.9 | -3.0 | 9.2 | 73 |  |
|  | | | | | |  |
| **2 day lag** | **All Children** | 1.9 | -1.9 | 5.8 | 169 |  |
|  | **No Allergy** | False Convergence | | | | p = 0.88 |
|  | **Presence of Any Allergy** | 3.1 | -2.0 | 8.4 | 116 |  |
|  | **No Dust Allergy** | -0.8 | -5.5 | 4.1 | 80 | p = 0.46 |
|  | **Dust Allergy** | 2.9 | -1.9 | 7.9 | 89 |  |
|  | **No Mould Allergy** | 1.6 | -2.2 | 5.5 | 139 | p = 0.40 |
|  | **Mould Allergy** | 3.7 | -16.7 | 28.9 | 30 |  |
|  | **No Pollen Allergy** | 2.7 | -1.5 | 7.1 | 101 | p = 0.52 |
|  | **Pollen Allergy** | False Convergence | | | |  |
|  | **No Fur Allergy** | 2.0 | -2.5 | 6.7 | 102 | p = 1.00 |
|  | **Fur Allergy** | False Convergence | | | |  |
